# Investigating the Antiparasitic Potential of the Marine Sesquiterpene Avarone, Its Reduced Form Avarol, and the Novel Semisynthetic Thiazinoquinone Analogue Thiazoavarone

**DOI:** 10.3390/md18020112

**Published:** 2020-02-14

**Authors:** Concetta Imperatore, Roberto Gimmelli, Marco Persico, Marcello Casertano, Alessandra Guidi, Fulvio Saccoccia, Giovina Ruberti, Paolo Luciano, Anna Aiello, Silvia Parapini, Sibel Avunduk, Nicoletta Basilico, Caterina Fattorusso, Marialuisa Menna

**Affiliations:** 1 The NeaNat Group, Department of Pharmacy, University of Naples “Federico II”, Via D. Montesano 49, 80131 Napoli, Italy; cimperat@unina.it (C.I.); marco.persico@unina.it (M.P.); marcello.casertano@unina.it (M.C.); pluciano@unina.it (P.L.); aiello@unina.it (A.A.); 2 Italian Malaria Network, Centro Interuniversitario di Ricerche Sulla Malaria (CIRM), Department of Pharmacy, University of Naples “Federico II”, Via D. Montesano 49, 80131 Napoli, Italy; 3 Institute of Biochemistry and Cell Biology, National Research Council, Campus A. Buzzati-Traverso, Via E. Ramarini, 32, 00015 Monterotondo (Roma), Italy; roberto.gimmelli@ibbc.cnr.it (R.G.); alessandra.guidi@ibbc.cnr.it (A.G.); fulvio.saccoccia@ibbc.cnr.it (F.S.); giovina.ruberti@cnr.it (G.R.); 4 Dipartimento di Scienze Biomediche per la Salute, Università di Milano, Via Pascal 36, 20133 Milan, Italy; silvia.parapini@unimi.it; 5 Department Vocational School of Medicinal Health Services, Mugla University, 48187 Mugla, Turkey; sibelavunduk@mu.edu.tr; 6 Dipartimento di Scienze Biomediche, Chirurgiche e Odontoiatriche, Università di Milano, Via Pascal 36, 20133 Milan, Italy; nicoletta.basilico@unimi.it

**Keywords:** *Dysidea avara*, avarone/avarol, redox-active compounds, quinones and hydroquinones, dioxothiazinoquinone, *Schistosoma mansoni*, *Plasmodium falciparum*, *Leishmania* spp., 3D-SAR analysis, DFT studies

## Abstract

The chemical analysis of the sponge *Dysidea avara* afforded the known sesquiterpene quinone avarone, along with its reduced form avarol. To further explore the role of the thiazinoquinone scaffold as an antiplasmodial, antileishmanial and antischistosomal agent, we converted the quinone avarone into the thiazinoquinone derivative thiazoavarone. The semisynthetic compound, as well as the natural metabolites avarone and avarol, were pharmacologically investigated in order to assess their antiparasitic properties against sexual and asexual stages of *Plasmodium falciparum*, larval and adult developmental stages of *Schistosoma* *mansoni* (eggs included), and also against promastigotes and amastigotes of *Leishmania infantum* and *Leishmania tropica*. Furthermore, in depth computational studies including density functional theory (DFT) calculations were performed. A toxic semiquinone radical species which can be produced starting both from quinone- and hydroquinone-based compounds could mediate the anti-parasitic effects of the tested compounds.

## 1. Introduction

Malaria and neglected tropical diseases (NTDs), a group of parasitic, bacterial, and viral infectious diseases (i.e., *Schistosoma* spp., *Leishmania* spp.), still have high morbidity and/or mortality rates worldwide. They affect more than one billion people and cause chronic illness, physical disability and/or deaths, especially in children and women of childbearing age, mostly in developing countries where they represent a serious hurdle to social and economic growth as well as a health problem [1,2]. Parasites belonging to different species can affect humans and animals concurrently, a phenomenon referred to as multiparasitism, which poses additional diagnostic and therapeutic challenges [3]. The therapeutic strategies for malaria and NTDs are very limited; drug-resistance phenomena, toxicity profiles and drug administration procedures of the few available chemical entities are still challenging. In this view, a research aimed to discover new chemicals active against several parasites is crucial and the marine environment may be an important resource [4,5]. In order to cope with all the reported drawbacks and to limit the costs of the development of brand-new pharmaceutical strategies, several effective antimalarial drugs should be considered for the treatment of other underfunded parasitic diseases. For example, artemisinin and its derivatives, a potent class of antimalarial agents, have been proved to be beneficial for other infectious diseases such as schistosomiasis and leishmaniasis [6]. Furthermore, histone deacetylases (HDAC) inhibitors have been shown to have activity both against some *Plasmodium* species as well as *Leishmania* and *Schistosoma* parasites [7]. Importantly, the blood parasites, *Plasmodium* and *Schistosoma*, both feeding on human hemoglobin, can detoxify the free heme groups through the synthesis of insoluble hemozoin pigments [8]. The interference with hemozoin formation featured an important antischistosomal mechanism of action showed by the antimalarial quinine and quinidine [9].

**Figure 1:**
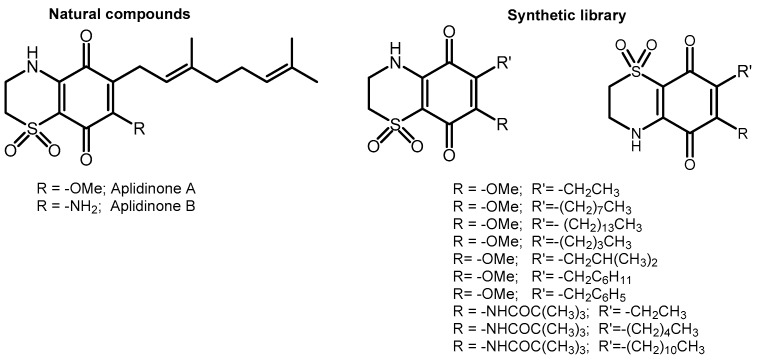
Structures of aplidinones A, B and of thiazinoquinone derivatives.

In the frame of our research for new anti-parasitic chemical entities, we recently identified the thiazinoquinone scaffold as a novel chemotype active against both *Plasmodium falciparum* and *Schistosoma mansoni* [10,11,12,13,14]. We developed this scaffold by creating of a chemical library of thiazinoquinone derivatives designed on the model of aplidinones, natural products isolated from a marine invertebrate (Figure 1). Many compounds exhibited in vitro antiplasmodial activities against the D10 and W2 strains of *P. falciparum* [11,13] with IC_50_ in the low micromolar range. Through an integrated experimental (cyclic voltammetry) and theoretical approach, we demonstrated that the antiplasmodial and anticancer activity of a series of thiazinoquinone compounds was not related to their two electrons redox potential [11,12]. In particular, the antiplasmodial activity was found to depend on the ability of the compound to generate a semiquinone radical species able to form a stable adduct with heme [11]. This was later supported by the design and synthesis of other sets of new thiazinoquinone derivatives, indicating that the activity was related to the ability to form a specific semiquinone radical, and to the ability of this latter to transfer the radical by and hydrogen-radical shift to the R substituent [13]. In addition, several important SARs were obtained. First, the thiazinoquinone moiety was ascertained to be necessary for the antiplasmodial activity, since the corresponding quinone derivatives (e.g., derivatives lacking the 1,1-dioxothiazine moiety) were inactive. Second, the regiochemistry of the heterocyclic ring with respect to the substituents (a methoxyl group and an alkyl chain) on the quinone ring was revealed as crucial for the activity. Third, the nature and shape of the R′ substituent were able to affect compound potency and selectivity.

Successively, we selected both some of the developed methoxy thiazinoquinones, and some ad hoc synthesized new derivatives with the aim of investigating the antischistosomal properties of this chemical scaffold. Compounds were thus tested against larval stage, adult worm couples and eggs of the platyhelminth *S. mansoni* [14]. Many of the tested molecules resulted active and, interestingly, as observed for the antiplasmodial activity, the effects against *S. mansoni* strongly depended on the regiochemistry of the heterocyclic ring, and from the nature and/or steric hindrance of the R′ substituent. Computational studies indicated that semiquinone radical species could be involved also in the mode of action against *S. mansoni* impairing the redox equilibrium within the parasite. Importantly, the R′ properties can affect both the pharmacodynamics and pharmacokinetics of the compounds [14].

**Figure 2:**
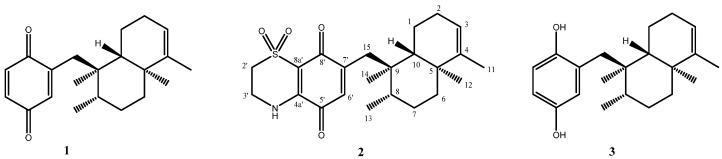
Structure of avarone (**1**), the semisynthetic thiazoavarone (**2**) and avarol (**3**).

In the course of a systematic chemical study of the macroflora and macrofauna of the coastal area of Turkey in the İzmir Bay (Aegean Sea), as a part of our ongoing search for bioactive marine-derived metabolites as leads for drug discovery [15,16,17,18,19,20], we isolated from the sponge *Dysidea avara* (Schmidt, 1862) the known sesquiterpene quinone avarone (**1**), along with its reduced form avarol (**3**, Figure 2) [21,22,23]. A wide range of pharmacological properties have been reported for the redox couple avarone (**1**) and avarol (**3**) including anti-tumor [24,25,26], anti-inflammatory [27,28,29], anti-mutagenic [30], anti-bacterial [31,32], anti-viral [33,34], anti-oxidant [23,35], anti-platelet [28], anti-psoriatic [36] and anti-biofouling [37,38] activities. Pharmacological studies on synthetic and semisynthetic derivatives of avarone have been previously reported, too [23,39,40,41]. Based on the above described extensive exploration of the thiazinoquinone scaffold as antiplasmodial and antischistosomal agent [11,13,14], we used the quinone avarone (**1**) as chemical starting point to obtain the semisynthetic thiazinoquinone derivative, thiazoavarone (**2**, Figure 2). Compound **2**, as well as the natural metabolites **1** and **3**, were investigated in order to evaluate their in vitro activity against: (i) asexual stages of D10 and W2 strains and stage V gametocytes of *P. falciparum*, (ii) egg production, larval and adult stages of *S. mansoni*, (iii) promastigote and amastigote stages of *Leishmania infantum* and *Leishmania tropica*. Computational studies, including density functional theory (DFT) calculations, were performed in order to analyze the conformational and redox properties of the two natural metabolites (**1** and **3**), as well as those of the novel semisynthetic analogue **2**. 

The obtained results shed light on the putative mechanism of action of the quinone/hydroquinone/thiazinoquinone compounds corroborating the hypothesis that their antiparasitic activity is related to the formation of a toxic semiquinone radical species. Noteworthy, thiazoavarone **2** resulted the most potent antimalarial thiazinoquinone developed by us, highlighting the important role for the activity played by the substituent of the 1,1-dioxo-1,4-thiazine ring. 

## 2. Results and Discussion

### 2.1. Chemistry

Avarone (**1**) and avarol (**3**) were isolated from the sponge *D. avara* and purified according to the previously described procedures [21,22,23]. They were easily identified by comparison of their spectroscopic properties (^1^H and ^13^C NMR, HRESI-MS) with those reported in literature [21,22,23].

**Scheme 1:**
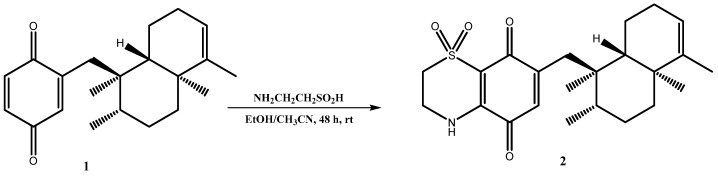
Coupling of avarone (**1**) with hypotaurine via nucleophilic addition reaction.

Thiazoavarone (**2**) was prepared as reported in Scheme 1. A portion of avarone was dissolved in a solution of CH_3_CN/EtOH (1:1) and then hypotaurine and a catalytic amount of salcomine in portion were added. The mixture was stirred for 48 h at room temperature and then was extracted with diethyl ether. The crude material was purified by HPLC on a reverse phase column (Luna 3 µm, 150 × 3.00 mm) (MeOH/H_2_O 75:25 v/v%) to afford the pure compound **2**. The nucleophilic addition reaction is regioselective in unsymmetrical quinones but generally leads to the formation of both regioisomers one of which is obtained in large excess with respect to the other. In the case of avarone, a single isomeric product, thiazoavarone **2**, has formed, as determined by MS and NMR spectroscopy; this could be reasonably due to the high steric hindrance of the heavy sesquiterpene moiety. 

**Figure 3:**
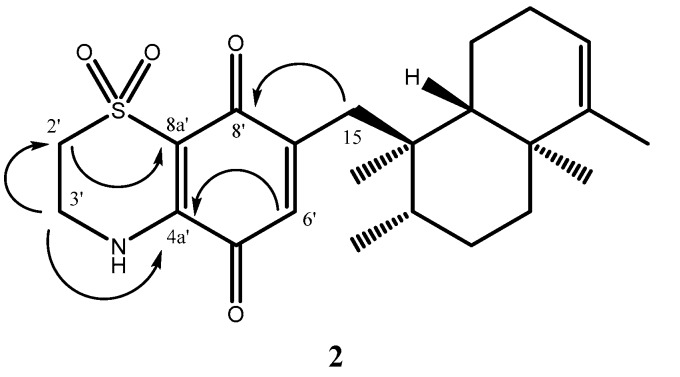
Key ^1^H-^13^C HMBC correlations of thiazoavarone (**2**).

Compound **2**, obtained as yellow powder, [α]_D_^25^ = +19.2 (c 0.004, MeOH), had a molecular formula of C_23_H_31_NO_4_S as determined by the HRESI MS ion at m/z C_23_H_31_NO_4_SNa [M + Na]^+^ 440.1865 (calculated value: 440.1866). Molecular formula obtained from MS and a first survey of the 1D NMR spectra of **2** (CDCl_3_) and the comparison with those of the known avarone **1** quickly allowed us to hypothesize that the condensation reaction with hypotaurine has occurred. ^1^H and ^13^C NMR data of **2** indicated the same decalin ring system of **1**, and the only difference between the two compounds is in the quinonic portion. Indeed, ^1^H NMR spectrum of **2** lacked the signals at δ_H_ 6.51 and 6.71, whereas contained a quite deshielded methylene signals (δ_H_3.30 and 4.05), resonating as two multiplets and each integrating for two protons. Likewise, the ^13^C spectrum of **2** contained two additional methilene carbon resonances at δ_C_ 39.8 and at δ_C_ 48.8 attributable to a nitrogen and sulfoxide-bearing carbons, respectively. The key HMBC cross-peaks (Figure 3) from H-3′ to C-2′ (δ_C_ 48.8), C-4a′ (δ_C_ 143.2), and from H-2′ to C-3′ (δ_C_ 39.8), C-8a′ (δ_C_ 111.6), from H-6′ to C-4a′ (δ_C_ 143.2), and from H-15a and H-15b′ to C-8′ (δ_C_ 177.1) indicated the regiochemistry of **2**. It should be noted that only one of possible regioisomer has been obtained. 

Chemical shifts and coupling patterns of the all signals of **2** were assigned by aid of COSY, HSQC, and HMBC experiments (Table 1). Anyway, a purity higher than 99.8% has been determined by HPLC for compounds **1**–**3**.

### 2.2. In Vitro Activity on P. falciparum and Cytotoxicity

Avarone (**1**) and the semisynthetic 1,1-dioxo-1,4-thiazine analogue (**2**), as well as hydroquinone avarol (**3**) were tested for their in vitro antiplasmodial activity against asexual and sexual (gametocytes stage V) stages of *P. falciparum* (Table 2). A chloroquine-sensitive (CQ-S) D10 and a chloroquine-resistant (CQ-R) W2 strains were used to determine the IC_50_ against asexual stage of parasites. The most potent compound was thiazoavarone (**2**) with an IC_50_ value in the nanomolar range, higher than those exhibited by the previously identified synthetic lead [13]. The high potency of the thiazinoquinone **2**, specifically on the chloroquine-resistant strain W2 and compared with avarone (**1**), lacking the heterocyclic moiety, confirmed once again the high potential of the thiazinoquinone scaffold for development of new antimalarial hits. Interestingly, both natural compounds **1** and **3** also exhibited a remarkable effect against sensitive and resistant *P. falciparum* strains, showing no cross-resistance with chloroquine (see Table 2). In particular, the reduced hydroquinone form (avarol, **3**) resulted significantly more active than the oxidized quinone form (avarone, **1**). 

The evaluation of the effects of compounds **1**–**3** on *Pf* gametocytes stage V, the sexual stage circulating in the bloodstream, was performed in order to evaluate their transmission blocking potential. As evidenced in Table 2, all the three compounds resulted less active against the gametocytes with respect to the parasite asexual stage; this finding is not unexpected since most of the current antimalarial drugs have no effect on the late stage of gametocytes. Avarol (**3**) resulted the most potent in the series against *Pf* gametocytes stage V with an IC_50_ = 9.30 μM, comparable to that of OZ27, a drug in clinical development (IC_50_ = 6.4 μM) [42]. 

Finally, we tested compounds **1**–**3** for their cytotoxic effects against two different human cell lines, microvascular endothelial (HMEC-1) and acute monocytic leukemia (THP-1) cells differentiated into macrophages; for each compound, we evaluated the selectivity index (SI, Table 2), namely the ratio between the IC_50_ on the human cells HMEC and that on the parasite strains (see Table 3). Thiazoavarone (**2**) exhibited a high toxicity against both mammalian cell lines, with IC_50_ in the low micromolar concentration range and, consequently, a very low SI. Avarone and avarol (**1** and **3**) were lowly and moderately cytotoxic, respectively (Table 3) but the hydroquinone **3** exhibited a better SI. 

### 2.3. In vitro Activity on Leishmania Parasites

To assess the antileishmanial activity of compounds **1**–**3** we tested them against promastigote stage of *L. infantum* and *L. tropica* responsible for visceral and cutaneous leishmaniasis, respectively. The results of this study are reported in Table 4 as IC_50_ values with the relevant selectivity indexes (SIs). Considering the couple avarone/avarol, we can notice that, as observed for the antiplasmodial effects, the reduced form (**3**) is substantially more potent than the oxidized form **1** on both investigated *Leishmania* parasites. This has been reported also for several antileishmanial naphthohydroquinones which resulted more active than the corresponding naphthoquinones [43]. Instead, the hydroquinone metabolite **3** and the thiazinoquinone **2** exhibited similar values of IC_50_ in the range of low micromolar; however, avarol (**3**) showed a significantly higher SI (see Table 4). Definitely, the above results displayed that introduction of the 1,1-dioxo-1,4-thiazine ring in the structure of avarone (**1**) to give thiazoavarone (**2**) meaningfully improves the antileishmanial activity, once again according to the antiplasmodial effects (see Table 2 and Table 4). 

In addition, compounds **1**–**3** were also investigated against intracellular amastigotes, the clinically relevant form of *Leishmania*. All compounds resulted from 2 to 4-fold more active against amastigotes than promastigotes; the hydroquinone avarol **3** confirmed itself as a promising agent to be further investigated in efficacy and selectivity, presenting the lowest IC_50_ value in the series and a SI greater than 10 (Table 4) [44].

### 2.4. In Vitro Activity on S. mansoni 

Compounds **1**–**3** were tested against larval stage (schistosomula), adult worm couples and eggs of the platyhelminth *S. mansoni*. The most potent compound on schistosomula was thiazoavarone (**2**) with a LC_50_ value in the low micromolar range (Table 5). The natural compounds (**1** and **3**) showed comparable activity, with avarol (**3**) slightly more active than avarone (**1**), both showing a LC_50_ in the high micromolar range (Table 5). Therefore, the presence of a thiazine ring was proved to be very important for activity on schistosomula. 

**Figure 4:**
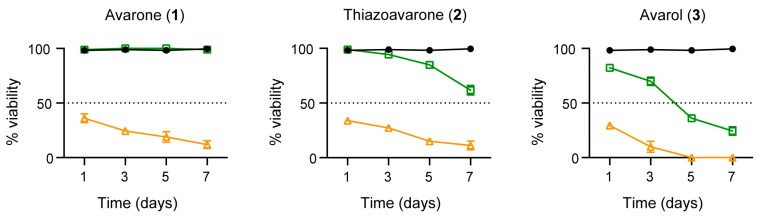
Compounds **1**–**3** impair adult *S. mansoni* viability. Worm pairs were incubated with DMSO (vehicle) (black circle) or the indicated compounds at 50 μM (yellow triangle), or 20 μM (green, square) as described in material and methods. Phenotype analysis was recorded for 7 days and % viability represents the mean ± SEM of three independent experiments.

All three compounds **1**–**3** were also very active on adult worm pairs at 50 μM leading to parasites death 7 days after treatment (Figure 4). However, when used at lower concentration (20 μM), only avarol (**3**) strongly impaired parasites viability (only 20% survival), while thiazoavarone (**2**) was poorly effective against the adult stage (Figure 4), despite its strong lethal effect on the larval stage (Table 5). These results suggest the possibility that the double lipid bilayer coating the adult worms, namely the tegument [45], can interfere with compound **2** uptake by adult parasites.

**Figure 5:**
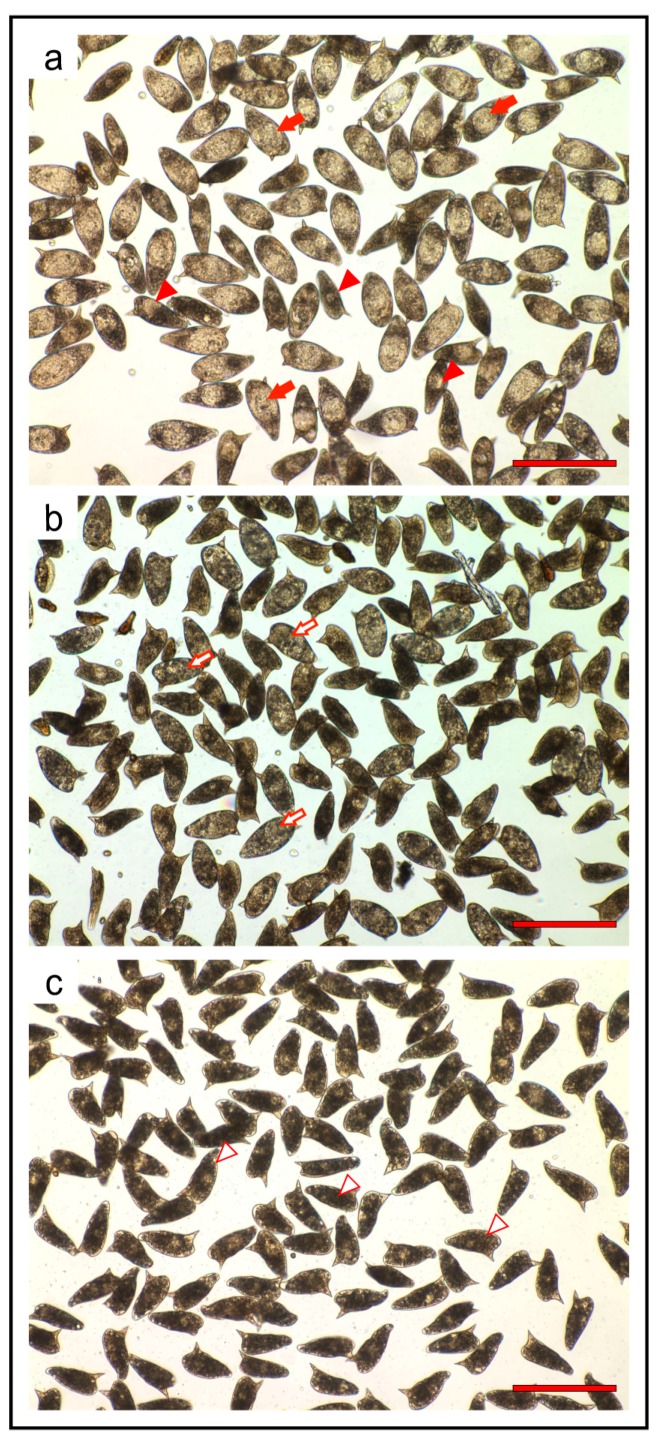
Thiazoavarone (**2**) impairs egg viability and maturation. Representative pictures of IVLEs treated with vehicle (DMSO) (**a**) or compound **2** at 5 μM (**b**) and 20 μM (**c**) for 72 h. Filled red arrows indicate viable eggs at stages III–V (intermediate/developed); filled red triangle indicate viable eggs at stages I–II (immature); red-edged arrows indicate damaged eggs at stages III–V; red-edged triangle indicate damaged eggs at stages I–II. Bar, 200 μm.

In the process of drug discovery for schistosomiasis, strategy involving any impairment in egg production and/or development must also be taken into account. In fact, upon mating with males, mature *S. mansoni* adult females, residing in the mesenteric veins of the definitive host, can lay hundreds of eggs each day. The eggs secreted in stool or trapped in the liver respectively cause disease transmission and, as a result of inflammatory granulomas reactions, intestinal and hepato-splenic diseases [46]. Therefore, compounds **1**–**3** were also assayed against the in vitro laid eggs (IVLEs). The IVLEs produced in the first 48 h by *S. mansoni* pairs and treated for 3 days with vehicle or compounds **1**–**3** were classified by microscopic observation according to the Vogel and Prata staging system of egg maturation [47]. The thiazoavarone (**2**) resulted the most effective compound, impairing eggs maturation already at 5 μM and resulting in undeveloped and severely damaged eggs at 20 μM (Figure 5). Similar results were obtained with compounds **1** and **3** at 50 μM.

### 2.5. Computational Studies and DFT Calculations

To rationalize the observed SARs, the steric and electronic features of compounds **1**–**3** were investigated by means of computational studies, including conformational analysis and DFT calculations.

A systematic conformational search considering all rotatable bonds was applied to generate all possible conformations of the compounds, which were, then, subjected to molecular mechanic (MM) geometry optimization using the CFF force field and a distance dependent dielectric constant value of 80 (Discovery Studio 2017, BIOVIA, San Diego USA; see the experimental Section for details) [48]. The global minimum energy conformer (GM) was identified for each compound. All the generated conformers presented an energy difference from the GM (Δ*E*_GM_) ≤ 3 kcal/mol. MM conformers were, then, subjected to density functional theory (DFT) calculations. In order to mimic an aqueous environment, all DFT calculations were performed using the conductor-like polarizable continuum model (C-PCM) as solvent model [49]. Moreover, to characterize every structure as minimum, a vibrational analysis was carried out (see the experimental Section for details). Fully optimized DFT conformers were classified into families according to the values of their torsion angles (Table 6 and Table 7 and Table S1). 

**Figure 6:**
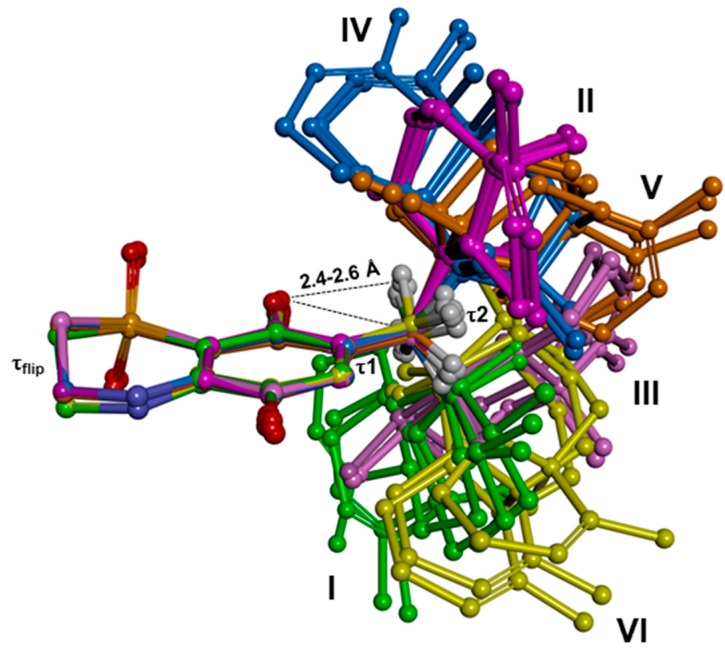
Density functional theory DFT conformers of compounds **1**–**3** superimposed by the carbon atoms of the quinone/hydroquinone ring. Carbon atoms are colored according to conformer classification (I = green, II = magenta, III = pink, IV = light blue, V = orange, and VI = Yellow); heteroatoms are colored by atom type (H = white, O = red, N = blue, S = orange). Hydrogens are omitted for sake of clarity, with the exception of those of the first methylene group of the R′ substituent, whose intramolecular distances from the nearby oxygen atom of the quinone are reported.

Results evidenced that compounds **1**–**3** present common conformational features, characterized by the electronic attraction between the hydrogen atoms of the first methylene group of the alkyl substituent and the nearby quinone oxygen, which limits the conformational freedom of R′ (Figure 6). Accordingly, the torsional angle τ1 showed just two sets of possible values (~±100°; Table 6 and Table 7 and Table S1), and for each of them the rigid sesquiterpene ring could assume three orientations with respect to the thiazinoquinone/quinone/quinol ring (τ2 = ~60°, ~−60° and ~180°; Table 6 and Table 7 and Table S1). This determined a total number of six conformers (named I–VI; Figure 6). In the case of the thiazinoquinone derivative **2**, due to the presence of the two opposite flips of the thiazinoquinone ring (τ_flip_ ~ ±60°), we obtained two specular sets of conformers with the same conformational energy (i.e., conformational enantiomers; Figure S10), as previously reported for other thiazinoquinone derivatives [13,14].

The fixed position of the methylene group combined with the presence of the rigid sesquiterpene moiety, place in the putative semiquinone radical produced upon one electron reduction/oxidation several hydrogen atoms at a distance (≤3 Å) suitable for an intramolecular radical shift from the oxygen atom to a carbon atom of R′ (see below). 

Then, starting from the DFT minima, the redox properties of **1**–**3** were calculated. At this aim, we considered the two electrons/two protons quinone reduction pathway in a protic solvent (Scheme S1) and all the species involved in the pathway (Q^•−^, QH^•^, QH^−^, QH_2_) were generated and DFT optimized using as starting structures the energetically favored DFT minima I and II. 

**Figure 7:**
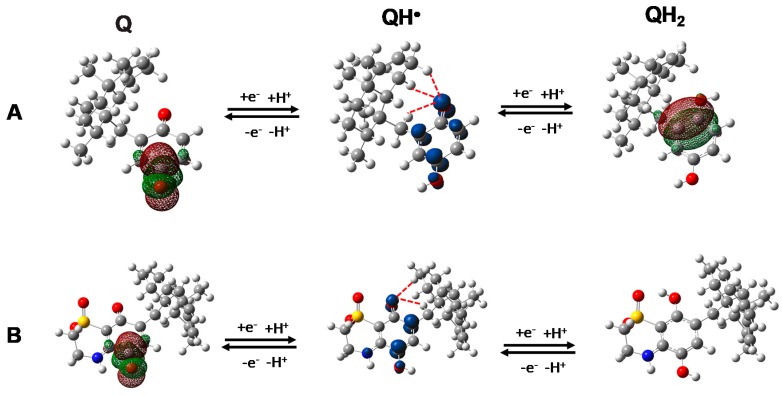
(**A**): DFT global minimum energy conformer (GM) structure of **1** (Q), DFT conformer I of **3** (QH_2_) and their semiquinone radical (QH^•^). (**B**) DFT GM structure of **2** (Q) together with its one- and two-electron reduced species QH^•^ and QH_2_. Atoms possibly involved in an intramolecular radical shift are evidenced with red dashed lines. The LUMO of **1** and **2**, and the HOMO of **3** are visualized using GaussView with an isosurface value of 0.02 e^−^/a.u.^3^ The NBO spin density isosurface of the QH^•^ species is displayed using GaussView with an isosurface value of 0.01 e^−^/a.u.^3^. The blue surface (positive spin density) corresponds to an excess of α-electron density.

Two possible protonated semiquinone species may be formed, depending on which of the two quinone oxygen atoms is reduced/oxidized at first. The location of the lowest unoccupied molecular orbital (LUMO) in **1** and **2** and of the highest occupied molecular orbital (HOMO) in **3** indicated the oxygen opposite to the alkyl chain as the most probable site to be reduced and the one close to the alkyl chain as the most probable site to be oxidized, respectively (Figure 7). It is worthy to be mentioned that, regarding the most probable oxidation pathway of **3** to its quinone form, since the deprotonation step is supposed to be the first event in protic solvents (Scheme S1), we calculated the pKa values of the two hydroxyl groups, too. Results indicated the hydroxyl group nearby the alkyl substituent as the first to lose the proton (Figure S11), further supporting the formation of the hydroquinone radical reported in Figure 7. 

The standard redox potential (E°) and the standard Gibbs free energy (ΔG^0^_red, aq_) of each electron-transfer reaction (see Scheme S1) alongside with the standard Gibbs free energy required for the protonation of the resulting reduced species (ΔG^0^_H+_) of the redox couple **1**, **3** and **2** were calculated (for details see the experimental Section). To further evaluate the propensity of **1** and **2** to undergo a one-electron reduction, the energy of the lowest unoccupied molecular orbital (E_LUMO_) was also taken into account. Similarly, we calculated the ionization potential (IP; i.e., −E_HOMO_) of the radical anion QH^−^ as indicative of the tendency of the deprotonated species of **3** to undergo a one-electron oxidation. Finally, we considered the energy of the single occupied molecular orbital (E_SOMO_) of the radical species as indicative of the ability to delocalize the unpaired electron. The resulting data are reported in Table 8 and Table 9. 

A first consideration can be derived comparing the redox properties of the quinone-based compounds **1** and **2**. With respect to **1**, **2** showed either a higher tendency to acquire one electron (lower E_LUMO_ and ΔG^0^_(red, aq)_; higher E°; Table 8) and a higher stability of the QH^•^ radical (lower E_SOMO_; Table 8). While the *E*_SOMO_ values of the anion radical species showed little difference (E_SOMO_ Q^•−^; Table 8), on the contrary, the differences became evident after the protonation step (E_SOMO_ QH^•^). These results, on one hand confirm the key role played by the 1,1-dioxo-1,4-thiazine ring on the electron affinity [11,13,14]; on the other hand, support the hypothesis that the compound activity is related to the formation of the semiquinone radical species. Indeed, the thiazinoquinone derivative **2** resulted overall more potent than the quinone derivative **1**. 

A second consideration is that, as evidenced in Figure 7 and Figure S12, all the calculated semiquinone radicals showed a hydrogen atom of the first methylene group together with, at least, another hydrogen atom of the rigid sesquiterpene ring, at a distance suitable for an intra-molecular hydrogen radical shift to the semi- reduced/oxidized oxygen atom (≤3 Å) (Table S2). By consequence, as above mentioned, the presence of the sesquiterpene moiety as alkyl substituent is expected to promote the putative “through space” intramolecular hydrogen radical shift leading to the formation of the toxic radical species. In line with this hypothesis, **2** resulted the most potent thiazinoquinone developed by us against *P. falciparum* D10 and W2 strains as well as against stage V gametocytes. In addition, **1**, although lacking the 1,1-dioxo-1,4-thiazine ring of **2**, resulted still active on *P. falciparum* D10 and W2 strains as well as on schistosomula, contrarily to what previously observed by us for other 1,4-benzoquinone derivatives when compared to the corresponding thiazinoquinone analogues [13,14]. 

Finally, the hydroquinone **3** showed a higher propensity to be oxidized to the semiquinone radical (i.e., lower E° and IP of the QH^−^ anion) with respect to the corresponding reduced form of **2** (Table 9). Thus, the absence of the 1,1-dioxo-1,4-thiazine ring favors the one-electron oxidation reaction of **3**. 

The hydroquinone **3** resulted more active than the corresponding quinone **1** against all considered parasites, while presenting the highest selectivity index with respect to mammalian cells. Both the oxidized and the reduced forms could produce the same putative toxic radical species upon a one-electron transfer reaction. In this view, our results suggest the presence in the parasite cell of a bioactivation reaction partner preferentially binding the hydroquinone (reduced) form (**3**) rather than the quinone (oxidized) form (**1**). This putative bioactivation partner seems not to be present in human cells. 

To investigate the role played by molecular pharmacokinetics on the observed SARs, we calculated the distribution coefficient values of **1**–**3** (clogD, Table S3) (ACD/Percepta 2017). According to Lipinski’s rules for drug absorption [50] the thiazinoquinone **2** showed a better cLogD value for cell membrane passive diffusion (cLogD ~ 4) with respect to **1** and **3** (cLogD > 5). However, in specific developmental stages (i.e., *Pf* late gametocytes, promastigote of *L. infantum* and *L. tropica*, amastigote of *L. infantum*, and adult worms of *S. mansoni*) **3** resulted more active than **2** (Table 2, Table 4 and Table 5; Figure 4 and Figure 5). According to what previously reported by us [14], this peculiar activity profile could be due to the significant morphological changes in the parasite during the above-mentioned developmental stages [51,52,53], which are likely to impair compound ability to penetrate into the parasite by passive diffusion, while it could still penetrate by exploiting the large number of transport proteins expressed on the parasite membrane. 

Taken together, the results of our computational investigation indicate that a toxic semiquinone radical species [11,13,14] which can be produced starting both from quinone- and hydroquinone-based compounds could mediate the anti-parasitic effects of the tested compounds **1**–**3**. 

## 3. Materials and Methods 

### 3.1. General Methods

Solvents: Carlo Erba (Pomezia, Rome, Italy). Commercial reagents: Sigma–Aldrich (Saint Louis, MO, USA). TLC: Silica Gel 60 F254, plates 5 × 20, 0.25 mm, Merck (Kenilworth, NJ, USA). Anhydrous solvents: Sigma-Aldrich-Merck. High-resolution ESI-MS analyses were performed on a Thermo LTQ Orbitrap XL mass spectrometer (Thermo-Fisher, San Josè, CA, USA). The spectra were recorded by infusion into the ESI (Thermo-Fisher, San Josè, CA, USA) source dissolving the sample in MeOH. ^1^H (500 MHz) and ^13^C (125 MHz) NMR spectra were recorded on an Agilent INOVA spectrometer (Agilent Technology, Cernusco sul Naviglio, Italy) equipped with a ^13^C enhanced HCN Cold Probe; chemical shifts were referenced to the residual solvent signal (CDCl_3_: δ_H_ = 7.26, δ_C_ = 77.0). For an accurate measurement of the coupling constants, the one-dimensional ^1^H NMR spectra were transformed at 64 K points (digital resolution: 0.09 Hz). Homonuclear (^1^H–^1^H) and heteronuclear (^1^H–^13^C) connectivities were determined by COSY and HSQC experiments, respectively. Two and three bond ^1^H-^13^C connectivities were determined by gradient 2D HMBC experiments optimized for a ^2,3^*J* of 8 Hz. ^3^*J*_H_–_H_ values were extracted from 1D ^1^H NMR. High performance liquid chromatography (HPLC) separations were achieved on a Shimadzu LC-10AT (Shimadzu, Milan, Italy) apparatus equipped with a Knauer K-2301 (LabService Analytica s.r.l., Anzola dell’Emilia, Italy) refractive index.

### 3.2. Collection, Extraction and Isolation

Several fresh specimens of *D. avara* were collected along the coast of Narlidere, Bay of Izmir (Turkey, 38°24′45 N 27°8′18 E), in summer of 2017 and immediately frozen and stored at −25 °C until the use. The identification of fresh material was performed by Mr. Arturo Facente while a voucher specimen is deposited at Mugla University, Turkey.

The freshly thawed sponge (21.9 g dry weight after extraction) was homogenized and treated at room temperature with methanol (3 × 1 L) and, subsequently, with dichloromethane (3 × 1 L). The combined extracts were concentrated in vacuo to give an aqueous suspension that was subsequently extracted with BuOH. The butanol soluble material (5.4 g of a dark brown oil), obtained after evaporation of the solvent, was chromatographed on a RP-18 silica gel flash column using a gradient elution (water→methanol→chloroform). The fractions eluted with H_2_O/MeOH 2:8 (*v*/*v*) were chromatographed by HPLC on an RP-18 column (Luna, 3 μm C-18, 150 × 3.00 mm), using MeOH/H_2_O 95:5 as the eluent (flow 0.5 mL/min). This separation afforded 4.5 mg of pure avarol (**3**, *t*_R_ = 5.4 min) and 26.8 mg of avarone (**1**, *t*_R_ = 9.4 min), identified by comparison of its spectral properties with literature values [21,22,23]. 

Avarone (**1**): yellow powder; [α]_D_^25^ = +2.6 (*c* 0.0014, CH_3_OH); ^1^H NMR (CDCl_3_) spectrum is reported in Supplementary Materials (Figure S6); HRMS (ESI): *m/z* 313.2156 [M + H]^+^ (calcd. for C_21_H_29_O_2_: 313.2162) (Figure S7). 

*Synthesis of thiazoavarone (**2**)*. 20.3 mg of avarone (**1**, 0.065 mmol) were dissolved in 12 mL of a mixture of CH_3_CN/EtOH 1: 1 (v/v) and kept under stirring at room temperature; then, a solution of hypotaurine (7 mg, 0.065 mmol) in 2 mL of water was added dropwise together with a catalytic amount of salcomine added in portions. The mixture was stirred for 48 h at room temperature before removing the most of ethanol in vacuo and pouring the residue into water. The orange/yellow mixture was extracted with diethyl ether (60 mL × three times) and the organic phase was washed with brine, dried over sodium sulfate, filtered, and solvent was removed by rotary evaporator. The resulting mixture was chromatographed by HPLC on an RP-18 column (Luna, 3 μm C-18, 150 × 3.00 mm) eluting with MeOH/H_2_O 75:25 (*t*_R_ = 31.6 min) and afforded the pure compound **2** (11 mg, 46%).

Thiazoavarone (**2**): orange powder; [α]_D_^25^ = +19.2 (*c* 0.0035, CDCl_3_); ^1^H and ^13^C NMR data are reported in Table 1. 1D and 2D NMR data, Figures S2–S5; HRMS (ESI): m/z 440.1865 [M + Na]^+^ (calcd. for C_23_H_31_NO_4_SNa: 440.1866) (Figure S1).

Avarol (**3**): yellow powder; [α]_D_^25^ = +12.4 (*c* 0.0018, CH_3_OH); ^1^H NMR (CDCl_3_) spectrum is reported in Supplementary Materials (Figure S8); HRMS (ESI): *m/z* 315.2337 [M + H]^+^ (calcd. for C_21_H_31_O_2_: 315.2319) (Figure S9).

### 3.3. P. falciparum Cultures and Drug Susceptibility Assay 

*P. falciparum* cultures were carried out according to Trager and Jensen with slight modifications [13]. The CQ-sensitive strain D10 and the CQ-resistant strain W2 were maintained at 5% hematocrit (human type A-positive red blood cells) in RPMI 1640 (EuroClone, Celbio) medium with the addition of 1% AlbuMax (Invitrogen, Milan, Italy), 0.01% hypoxanthine, 20 mM Hepes, and 2 mM glutamine at 37 °C in a standard gas mixture (1% O_2_, 5% CO_2_, and 94% N_2_). All compounds were dissolved in DMSO and then diluted with medium to achieve the required concentrations (final DMSO concentration <1%, non-toxic to the parasite). Drugs were placed in 96-well flat-bottomed microplates and serial dilutions made. Asynchronous cultures with parasitaemia of 1%–1.5% and 1% final hematocrit were aliquoted into the plates and incubated for 72 h at 37 °C. Parasite growth was determined spectrophotometrically (OD650) by measuring the activity of the parasite lactate dehydrogenase (pLDH), according to a modified version of the method of Makler in control and drug-treated cultures [13]. The antiplasmodial activity is expressed as 50% inhibitory concentrations (IC_50_); each IC_50_ value is the mean ± standard deviation of at least three separate experiments performed in duplicate. 

### 3.4. Gametocytes Cultivation and Susceptibility Assay

The transgenic *P. falciparum* 3D7 strain 3D7elo1-pfs16-CBG99 expressing the *Pyrophorus plagiophthalamus* CBG99 luciferase under a gametocyte specific promoter was used in all the experiments. Parasites were cultured and gametocytes obtained as previously described [54]. Late-stage gametocytes were exposed to compounds at day 11 after *N*-acetylglucosamine (NAG) addition. Gametocytes stages were counted in Giemsa stained smears and the percentage of stage V gametocytes was higher than 80%. Compounds were prepared by serial dilution, in 96-well plate, in complete medium. Plates were incubated for 72 h at 37 °C under 1% O_2_, 5% CO_2_, 94% N_2_ atmosphere. Luciferase activity was taken as measure of gametocytes viability, as previously described [55]. Briefly, drug-treated gametocyte samples at 2% haematocrit were transferred to 96-well black microplates and d-luciferin (1 mM in citrate buffer 0.1 M, pH 5.5) was added at a 1:1 volume ratio. Luminescence measurements were performed after 10 min with 500 ms integration time using a Sinergy 4 (Biotek) microplate reader. The IC_50_ was extrapolated from the non-linear regression analysis of the concentration–response curve.

### 3.5. In Vitro Promastigote Susceptibility Assays

Promastigote stage of *L. infantum* strain MHOM/TN/80/IPT1 and *L. tropica* (MHOM/IT/2012/ISS3130) were cultured in Schneider’s Drosophila medium (Lonza) supplemented with 10% heat-inactivated fetal calf serum (HyClone) at 24 °C.

The complete medium used for antileishmanial activity assay was RPMI (EuroClone) supplemented with 10% heat-inactivated fetal calf serum (EuroClone), 20 mM Hepes, and 2 mM l-glutamine. To estimate the 50% inhibitory concentration (IC_50_), the MTT (3-[4.5-dimethylthiazol-2-yl]-2.5-diphenyltetrazolium bromide) method was used. Compounds were dissolved in DMSO and then diluted with medium to achieve the required concentrations. Drugs were placed in 96-well round-bottom microplates and seven serial dilutions made. Amphotericin B was used as the reference anti-leishmanial drug. Parasites were diluted in complete medium to 5 × 10^6^ parasites/mL and 100 μL of the suspension was seeded into the plates, incubated at 24 °C for 72 h and then 20 µL of MTT solution (5 mg/mL) was added into each well for 3 h. The plates were then centrifuged, the supernatants discarded and the resulting pellets dissolved in 100 µL of lysing buffer consisting of 20% (*w*/*v*) of a solution of SDS (Sigma), 40% of *N*,*N*-dimethylformamide (Merck) in H_2_O. The absorbance was measured spectrophotometrically at a test wavelength of 550 nm and a reference wavelength of 650 nm. The results are expressed as IC_50_ which is the dose of compound necessary to inhibit parasite growth by 50%; each IC_50_ value is the mean ± standard deviation of separate experiments performed in duplicate.

### 3.6. In Vitro Intracellular Amastigote Susceptibility Assays

THP-1 cells (human acute monocytic leukemia cell line) were maintained in RPMI supplemented with 10% FBS (EuroClone), 50 μM 2-mercaptoethanol, 20 mM Hepes, 2 mM glutamine, at 37 °C in 5% CO_2_. For *Leishmania* infections, THP-1 cells were plated at 5 × 10^5^ cells/mL in 16-chamber Lab-Tek culture slides (Nunc) and treated with 0.1 μM phorbol myristate acetate (PMA, Sigma) for 48 h to achieve differentiation into macrophages. Cells were washed and infected with metacyclic *L. infantum* promastigotes at a macrophage/promastigote ratio of 1/10 for 24 h. Cell monolayers were then washed and incubated in the presence of test compounds for 72 h. Slides were fixed with methanol and stained with Giemsa. The percentage of infected macrophages in treated and non-treated cells was determined by light microscopy.

### 3.7. Cytotoxicity Assay

The long-term human microvascular endothelial cell line (HMEC-1) was maintained in MCDB 131 medium (Invitrogen, Milan, Italy) supplemented with 10% fetal calf serum (HyClone, Celbio, Milan, Italy), 10 ng/mL of epidermal growth factor,1 µg/ml of hydrocortisone, 2 mM glutamine and 20 mM Hepes buffer (EuroClone). For the cytotoxicity assays, HMEC-1 were plated at 10^5^ cells/mL in 96-well flat bottom microplates. THP-1 cells were plated at 5 × 10^5^ cells/mL in 96-well flat bottom microplates and treated with 0.1 μM PMA for 48 h to achieve differentiation into macrophages. Cells were then treated with serial dilutions of test compounds and cell proliferation evaluated using the MTT assay described for promastigotes. The results are expressed as IC_50_, which is the dose of compound necessary to inhibit cell growth by 50%.

### 3.8. In vitro Effects of Compounds on S. mansoni Parasites and Eggs

Schistosomula were prepared by mechanical transformation of cercariae, as previously described [56]. The ATP-based viability assay with CellTiterGlo (Promega, Italy) on the larval stage of schistosomes was carried out in 96-well, black, tissue culture plates by adaptation of a protocol previously set up in our laboratory [56]. DMSO (vehicle) and gambogic acid (10 μM) were used as the negative and positive controls in each plate and the percentage of viability for each compound was calculated as the ATP reduction against vehicle (0%) and gambogic acid (100%).

All Animal work was approved by the National Research Council, Institute of Cell Biology and Neurobiology animal welfare committee (OPBA) and by the competent authorities of the Italian Ministry of Health, DGSAF, Rome (authorization no. 25/2014-PR and no. 336/2018-PR). All experiments were conducted in respect to the 3R rules according to the ethical and safety rules and guidelines for the use of animals in biomedical research provided by the relevant Italian law and European Union Directive (Italian Legislative Decree 26/2014 and 2010/63/EU) and the International Guiding Principles for Biomedical Research involving animals (Council for the International Organizations of Medical Sciences, Geneva, Switzerland).

A Puerto Rican strain of *S. mansoni* was maintained by passage through albino *Biomphalaria glabrata*, as the intermediate host, and ICR (CD-1) outbred female mice as previously described [56]. Female 4 to 7-week-old mice (Envigo, Udine, Italy) were infected with 200–400 double sex *S. mansoni* cercariae by the tail immersion technique. Adult pairs were harvested from mice 7–8 weeks after infection by reversed perfusion of the hepatic portal system and mesenteric veins. For the viability assays, 5 couples were incubated with the compounds in 3 mL DMEM complete tissue culture medium containing 10% FBS for up to 7 days and phenotypic score was assigned as previously described [57]. For egg-treatment, 5 worm pairs were incubated for 48 h in 3 mL complete tissue culture medium; next the parasites were removed and vehicle (DMSO) or compounds **1**–**3** were added to each plate containing the eggs previously laid in vitro and observed for 72 h as previously reported [14]. Briefly, images were recorded with a BX41 Olympus microscope and a bright field objective 10× served by a SPOT RT 220-3 Diagnostic Instrument Inc camera. Egg maturation/morphological score was assigned based on the Vogel and Prata’ staging system of egg maturation [47]. 

### 3.9. Molecular Modelling

Molecular modelling calculations were performed on E4 Server Twin 2× Dual Xeon-5520, equipped with two nodes. Each node: 2× Intel^®^ Xeon^®^ QuadCore E5520-2.26Ghz, 36 GB RAM. The molecular modelling graphics were carried out on a personal computer equipped with Intel(R) Core(TM) i7-4790 processor and SGI Octane 2 workstations. 

*Conformational property analysis.* The apparent pKa and logD values (pH 7.4 and 7.2) of compounds were calculated by using the ACD/Percepta software (ACD/Percepta, Advanced Chemistry Development, Inc., Toronto, ON, Canada, 2017, http://www.acdlabs.com). 

The compounds were considered neutral in all calculations performed as a consequence of the estimation of percentage of neutral/ionized forms computed at pH 7.4 (blood pH value) and pH 7.2 (cytoplasm pH value) using the Henderson–Hasselbalch equation. The compounds were built using the Small Molecule tool of Discovery Studio 2017 (Dassault Systèmes BIOVIA, San Diego). Then, the built compounds were subjected to molecular mechanic (MM) energy minimization (ε = 80 × *r*) until the maximum RMS derivative was less than 0.001 kcal/Å, using Conjugate Gradient [58] as minimization algorithm. Atomic potentials and charges were assigned using the CFF forcefield [48]. The conformers obtained for each compound were used as starting structure for the subsequent systematic conformational analysis (Search Small Molecule Conformations; Discovery Studio 2017). The conformational space of the compounds was sampled by systematically varying the rotatable bonds sp^3^–sp^3^ and sp^3^–sp^2^ with an increment of 60°. The RMSD cutoff for structure selection was set to 0.01 Å. Finally, to ensure a wide variance of the input structures to be successively fully minimized, an energy threshold value of 10^6^ kcal/mol was used as selection criteria. The generated structures were then subjected to MM energy minimization (CFF forcefield; ε = 80 × *r*) until the maximum RMS derivative was less than 0.001 kcal/Å, using Conjugate Gradient as minimization algorithm. Finally, the resulting conformers were ranked by their potential energy values (i.e., ΔE from the global energy minimum (GM)).

All MM conformers within 5 kcal/mol from GM has been then subjected to DFT calculations. The calculations were carried out using the Gaussian 09 package [59]. All structures were fully optimized at the B3LYP/6-31+G (d,p) level using the conductor-like polarizable continuum model (C-PCM) [49,60,61]. In order to characterize every structure as minimum and to calculate the Gibbs free energy a vibrational analysis was carried out at the same level of theory using the keyword: freq. The RMS force criterion was set to 3 × 10^−4^ a.u. Partial charges, molecular orbitals and spin density have been calculated using the natural bond orbital (NBO) method [62]. The resulting conformers were ranked by their potential energy values (i.e., ΔE from the global energy minimum (GM)) and classified by their dihedral angle values).

**Scheme 2:**
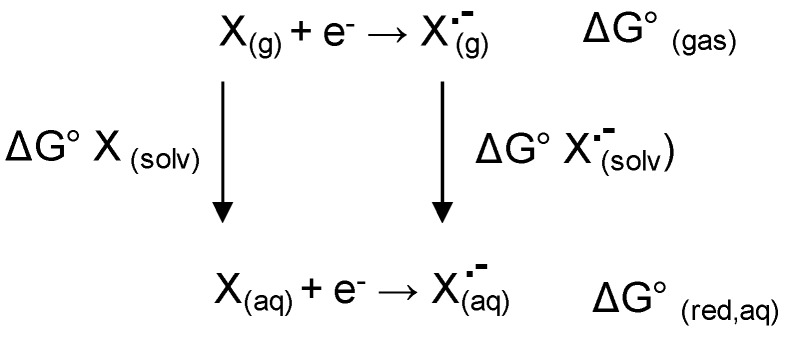
Born–Haber cycle for a generic one-electron transfer reaction in vacuo and in aqueous solution.

*Calculation of redox properties.* An appropriate way for calculating the redox potential is by using a thermodynamic cycle. Accordingly, the redox potentials for the compounds was calculated by using the Born–Haber cycle (Scheme 2), which links the Gibbs free energy change of the one-electron transfer reaction in the gas phase with that of the same reaction in aqueous solution [63,64].

Accordingly, the Born–Haber thermodynamic cycle allows to include the desolvation/solvation effects by calculating the Gibbs free energy values of the reaction in gas phase and in solution. According to this approach, the standard Gibbs free energy of the electron transfer ($\Delta G_{red,aq}^{0}$), was calculated taking into account the free energy change in the gas phase ($\Delta G_{gas}^{0})\;$ and the solvation free energies of the oxidized ($\Delta G_{solv}^{0}\left(X\right))\;$ and reduced species $(\Delta G_{solv}^{0}\left(X^{•-})\right)$ as reported in the following equation:

(1) $$\Delta G_{red,aq}^{0}=\Delta G_{gas}^{0}+(\Delta G_{solv}^{0}\left(X^{•-}\right)-\Delta G_{solv}^{0}\left(X\right))$$

Accordingly, we, firstly, calculated the Gibbs free energy in gas phase by using the following equation:

(2) $$\Delta G_{gas}^{0}=G_{gas}^{0}\left(X^{•-}\right)-G_{gas}^{0}\left(X\right)$$

Then, the Gibbs free energy of solvation of both the species X and X^•−^ were calculated according to the following equations:

(3) $$\Delta G_{solv}^{0}\left(X\right)=G_{aq}^{0}\left(X\right)-G_{gas}^{0}\left(X\right)$$

(4) $$\Delta G_{solv}^{0}\left(X^{-}\right)=G_{aq}^{0}\left(X^{•-}\right)-G_{gas}^{0}\left(X^{•-}\right)$$

The values obtained were used to calculate the standard Gibbs free energy of the overall reaction ($\Delta G_{red,aq}^{0}$; kcal·mol^−1^) according to Equation (1). The X and X^•−^ species correspond in our case to Q and Q^•−^, respectively in the first electron transfer, and to QH^•^ and QH^−^ in the second electron transfer (see Scheme S1). 

Finally, the $\Delta G_{red,aq}^{0}$, were used to calculate the standard reduction potential by the Nernst equation:

(5) $$E^{0}\;=\;-\frac{△G_{red,aq}^{0}}{nF}$$

where *n* is the number of electrons involved in the process (i.e., 1) and *F* the Faraday constant (23.06 kcal mol^−1^ V^−1^). 

The reduction potential given by the Equation (5) is an absolute value of the parameter and has to be referred to the standard hydrogen electrode (SHE; redox potential of 4.43 V), to obtain the standard redox potential. Accordingly, the value of 4.43 V was subtracted from the values obtained by Equation (5). 

On these bases, in order to calculate the redox potential, starting from the structure of the DFT Q GM conformers (i.e., the starting quinone species), the redox states Q^•−^, QH^•^, QH^−^, QH_2_ were generated. Following the reduction pathway of quinones showed in Scheme S1, each species was generated starting from the DFT optimized species of the previous step and submitted to full DFT optimization both in aqueous solution (using the C-PCM method) and in vacuo using the same parameters above described. 

The standard Gibbs free energy for the protonation of the reduced species were calculated by determining ΔG° of the reaction Y + H^+^ → YH that is ΔG° = G°(YH) − G°(Y), where Y is Q^•−^ or QH^−^ DFT conformer. The energy of the frontier molecular orbitals (HOMO, LUMO, and SOMO) were considered for the species Q, Q^•−^, QH^•^ and QH^−^ fully optimized by DFT calculations performed in the aqueous solution (using the C-PCM method). According to the Koopmans’ theorem [65], the ionization potential (IP) is given by the negative of the value of the energy of the HOMO.

## 4. Conclusions

We investigated the antiparasitic potential of the marine sesquiterpene avarone (**1**), its reduced form avarol (**3**), and thiazoavarone (**2**), a novel semisynthetic thiazinoquinone analogue obtained through a condensation reaction of **1** with hypotaurine, which resulted completely regioselective**.** Both the natural metabolites **1** and **3**, as well as the semisynthetic derivative **2** resulted active against D10 and W2 strains and late stage gametocytes of *P. falciparum*, against larval and adult developmental stages, and eggs of the platyhelminth *S. mansoni*, and also against promastigote and amastigote forms of *L. infantum* and *L. tropica*. The observed differences in the magnitude of the effects of the three molecules allowed us to draw some interesting conclusions, strongly supported by the results of the DFT analysis. In particular, the thiazinoquinone **2** resulted as significantly more potent than the quinone derivative **1** against D10 and W2 strains of *P. falciparum* as well as on the larval stage of *S. mansoni*. On the other hand, compound **1**, although lacking the 1,1-dioxo-1,4-thiazine ring, resulted still active on *P. falciparum* and on *Schistosoma* larval stage, contrarily to what previously observed for other 1,4-benzoquinone derivatives when compared to the corresponding thiazinoquinone analogues [13,14]. The calculated redox properties of **1** and **2** evidenced for **2** either a higher tendency to acquire one electron and a higher stability of the QH^•^ radical. Noteworthy, thiazoavarone **2** resulted the most potent antimalarial thiazinoquinone developed by us.

The hydroquinone **3** resulted more active than the corresponding quinone **1** against all considered parasites, with the highest selectivity index with respect to mammalian cells. This led to hypothesize the presence in the studied parasites of a bioactivation reaction partner preferentially binding the reduced form (**3**) rather than the oxidized form (**1**). This putative bioactivation partner seems not to be present in human cells. Finally, comparison of the standard redox potential (E°) and of the ionization potential (IP) of the QH^-^ anion of **3** with those of the corresponding reduced form of **2**, indicated a higher propensity of **3** to be oxidized to the semiquinone radical and, thus, that the absence of the 1,1-dioxo-1,4-thiazine ring favours the one-electron oxidation reaction of **3**.

These results confirm the key role played by the 1,1-dioxo-1,4-thiazine ring on the electron affinity [11,13,14] and, mainly, corroborate the hypothesis that the compound activity is related to the formation of a toxic semiquinone radical species, which can be produced, upon a one-electron transfer reaction, starting both from quinone- and hydroquinone-based compounds. In the case of the antiplasmodial activity (on the erythrocytic stage of the parasite), our previous results indicated that the bioactivation partner can be represented by free heme (generated during hemoglobin digestion) [11,13]. On the contrary, in the case of *S. mansoni* as well as *Leishmania* parasites, at this stage, we do not know the bioactivation partner of the compounds. However, reactive oxygen species (ROS) generation was found to be involved in the pro-apoptotic mechanism of some natural marine derived thiazinoquinones against Jurkat cells [66]. Thus, it cannot be ruled out that the putative semiquinone radical species reacts with oxygen molecules in cells, generating ROS and future studies will be devoted to clarifying this issue. Moreover, the observed difference between the activity trend of **1**–**3** against the different parasites and their developmental stage may be related to morphological and/or metabolic differences in the targeted organism/stage. Thus, the selective toxicity against the different parasites and their developmental stages can be addressed, taking advantage of the possible bioactivation reaction partners to form the putative toxic radical (i.e., one-electron reduction or oxidation reaction).

## Figures and Tables

**Table 1:** ^1^H (700 MHz) and ^13^C (125 MHz) NMR data of thiazoavarone (**2**) in CDCl_3_.

Pos.	δ_C_	δ_H,_ mult. (*J* in Hz)	Pos.	δ_C_	δ_H_, mult. (*J* in Hz)
**1** **′**	-	-	**4**	143.7	-
**2** **′**	48.8	3.30, m	**5**	38.3	-
**3** **′**	39.8	4.05, m	**6**	35.8	1.03 ^a^, 1.64 ^a^
**4** **′**	-	6.41, br s	**7**	27.3	1.38 ^a^
**4a** **′**	143.2	-	**8**	37.1	1.25 ^a^
**5** **′**	179.3	-	**9**	43.7	-
**6** **′**	131.8	6.50, s	**10**	47.5	1.03 ^a^
**7** **′**	152.8	-	**11**	17.9	1.53, s
**8** **′**	177.1	-	**12**	20.0	0.99, s
**8a** **′**	111.6	-	**13**	16.7	0.96, d, (6.2)
**1**	19.3	1.50 ^a^, 1.85, dd, (6.7, 13.2)	**14**	17.7	0.85, s
**2**	26.3	1.93 ^a^, 2.04 ^a^	**15a**	35.6	2.50, d, (13.1)
**3**	120.9	5.2, br s	**15b**		2.70, d, (13.1)

^a^ Overlapped by other signals.

**Table 2:** In vitro antimalarial activity against asexual *P. falciparum* parasites from D10 (CQ-sensitive) and W2 (CQ-resistant) strains ^a^ and against stage V *P. falciparum* gametocytes from a 3D7 transgenic line.

Compounds	D10 (μM) ^b^	W2 (μM) ^b^	*Pf* Gametocytes Stage V 3D7elo1-pfs16-CBG99 IC_50_ (μM) ^b^
Avarone (**1**)	2.74 ± 0.51	2.09 ± 0.52	15.53 ± 5.26
Thiazoavarone (**2**)	0.38 ± 0.15	0.21 ± 0.03	15.01 ± 3.19
Avarol (**3**)	0.96 ± 0.24	1.10 ± 0.15	9.30 ± 1.90
Methylene blue	-	-	0.155 ± 0.05

^a^ Chloroquine (CQ) has been used as positive control (D10 IC_50_ = 0.04 ± 0.01; W2 IC_50_ = 0.54 ± 0.28). ^b^ The results are the mean ± SD of IC_50_ of three independent experiments performed in duplicate.

**Table 3:** IC_50_ against HMEC-1 (human microvascular endothelial cells) and THP-1 (human acute monocytic leukemia cells) and Selectivity Index (SI) of the compounds **1**–**3**.

Compounds	HMEC-1 IC_50_ (μM) ^a,b^	THP-1 IC_50_ (μM) ^a,c^	SI ^d^	
			D10	W2
Avarone (**1**)	62.19 ± 1.98	>100	22.7	29.8
Thiazoavarone (**2**)	3.31 ± 1.53	7.41	8.7	15.8
Avarol (**3**)	36.85 ± 5.79	31.75	38.4	33.5

^a^ Camptothecin has been used as positive control (IC_50_ (μM) = 0.018 ± 0.008 on HMEC-1). ^b^ Data are expressed as mean ± SD of three different experiments performed in duplicate. ^c^ Data are the mean of two different experiments in duplicate. ^d^ SI = IC_50_ HMEC-1/IC_50_ *P. falciparum* strain.

**Table 4:** Activity of compounds **1**–**3** against promastigote stage of *L. infantum* and *L. tropica* and against amastigote stage of *L. infantum.*

Compounds	*L. infantum* IC_50_ (μM) ^a^	SI_p_^c^	*L. tropica* IC_50_ (μM) ^a^	SI_p_^c^	*L. infantum* Amastigotes IC_50_ (μM) ^b^	SI_a_^d^
Avarone (**1**)	28.21 ± 0.32	2.2	20.28 ± 3.56	3.1	7.64	8.1
Thiazoavarone (**2**)	8.78 ± 0.26	0.38	9.52 ± 0.32	0.35	4.99	0.67
Avarol (**3**)	7.42 ± 0.27	5.0	7.08 ± 1.91	5.2	3.19	11.6
Amphotericin B	0.20 ± 0.03		0.17 ± 0.04		0.189	

^a^ Data are expressed as mean ± SD of three different experiments performed in duplicate. ^b^ Data are the mean of two different experiments in triplicate. ^c^ SI_p_ = IC_50_ HMEC-1/IC_50_ *L. infantum* (*L. tropica*) promastigotes. ^d^ SI_a_ = IC_50_ HMEC-1/IC_50_ L. infantum amastigotes.

**Table 5:** Activity of compounds **1**–**3** against *S. mansoni* schistosomula.

Compounds	LC_50_ (μM) ^a^
Avarone (**1**)	42.77 ± 1.90
Thiazoavarone (**2**)	5.90 ± 2.59
Avarol (**3**)	33.97± 5.52

^a^ Data are expressed as mean ± SD of three different experiments.

**Table 6:**
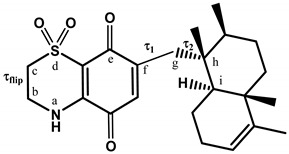
ΔE_GM_ values (kcal/mol) and torsion angle values (degrees) of the DFT conformers of **2**.

Conformer ^a^	ΔE_GM_ (kcal/mol)	Torsion Angles (°)	
		τ1 ^b^	τ2 ^c^
**II**	0.00	100.38	60.20
**I**	0.50	−110.21	55.83
**IV**	1.04	110.37	−72.73
**III**	1.25	−102.31	179.90
**V**	2.03	87.13	158.72
**VI**	3.02	−84.08	−54.03

^a^ Only the conformational enantiomers with the value of τ_flip_ ~60° are reported. ^b^ τ1 torsion angle is defined by e, f, g, and h atoms. ^c^ τ2 torsion angle is calculated considering f, g, h, and i atoms.

**Table 7:**
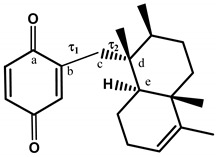
ΔE_GM_ values (kcal/mol) and torsion angle values (degrees) of the DFT conformers of **1**.

Conformer	ΔE_GM_	Torsion Angles	
	(kcal/mol)	(°)	
		τ1 ^a^	τ2 ^b^
**I**	0.00	−91.95	64.65
**II**	0.01	101.16	60.62
**III**	1.20	−92.79	169.87
**IV**	1.25	96.73	−58.29
**V**	2.33	84.62	173.14
**VI**	2.64	−81.09	−54.96

^a^ τ1 torsion angle is defined by a, b, c, and d atoms. ^b^ τ2 torsion angle is calculated considering b, c, d, and e atoms.

**Table 8:** DFT calculated parameters and standard redox potentials (E°; Q/Q^•−^) of compounds **1**–**3**.

Cmp.	Conf.	E_LUMO_^a^	E_SOMO_^a^	E_SOMO_^a^	ΔG°_red, aq_^a^	E° ^b^	ΔG°_H+_^a^
		(Q)	(Q^•−^)	(QH^•^)	(Q/Q^•−^)	(Q/Q^•−^)	(Q^•−^/QH^•^)
**1, 3**	I	−10.62	−143.34	−106.45	−94.58	−328.57	−279.60
**1, 3**	II	−10.68	−143.42	−106.60	−95.13	−304.65	−279.87
**2**	I	−17.76	−148.69	−158.60	−95.29	−297.63	−272.91
**2**	II	−17.83	−148.72	−159.93	−95.66	−281.68	−273.03

^a^ kcal/mol. ^b^ mV.

**Table 9:** DFT calculated parameters and standard redox potentials (E°; QH^•^/QH^−^) of compounds **1**–**3**.

Cmp.	Conf.	ΔG°_red,aq_^a^	E° ^b^	ΔG°_H+_^a^	IP ^a^	E_HOMO_^a^
		(QH^•^/QH^−^)	(QH^•^/QH^−^)	(QH^−^/QH_2_)	(QH^−^)	(QH_2_)
**1, 3**	I	−91.67	−454.64	−295.41	61.09	−158.06
**1, 3**	II	−89.95	−529.31	−296.77	60.83	−156.34
**2**	I	−95.22	−300.60	−290.49	130.87	−158.18
**2**	II	−95.24	−299.70	−289.55	130.94	−159.27

^a^ kcal/mol. ^b^ mV.

## Supplementary Materials

The following are available online at https://www.mdpi.com/1660-3397/18/2/112/s1, Figure S1. HRESIMS spectrum of thiazoavarone (**2**), Figure S2. 1H NMR spectrum of thiazoavarone (**2**) in CDCl3, Figure S3. 1H-1H COSY spectrum of thiazoavarone (**2**) in CDCl3, Figure S4. HSQC spectrum of thiazoavarone (**2**) in CDCl3, Figure S5. HMBC spectrum of thiazoavarone (**2**) in CDCl3, Figure S6. HRESIMS spectrum of avarone (**1**), Figure S7. 1H-NMR spectrum of avarone (**1**) in CDCl3, Figure S8. HRESIMS spectrum of avarol (**3**), Figure S9. 1H-NMR spectrum of avarol (**3**) in CDCl3, Figure S10. Conformational enantiomers of thiazoavarone (**2**), Figure S11. Calculated pka values of avarol (**3**) (ACD/Percepta software), Figure S12. DFT conformer of **1**-**3**, and their radical species, Table S1. ΔEGM values and torsion angle values of the DFT conformers of avarol (**3**), Table S2. Hydrogen atoms suitable for an intramolecular radical shift, Table S3. cLogD values of **1**–**3**, Scheme S1. Reduction pathway of quinones in a protic solvent.
