# Supplementary material for: Investigating the Antiparasitic Potential of the Marine Sesquiterpene Avarone, Its Reduced Form Avarol, and the Novel Semisynthetic Thiazinoquinone Analogue Thiazoavarone
Source: Mar Drugs. 2020 Feb 14;18(2):112. doi: 10.3390/md18020112 (PMC7074381; doi:10.3390/md18020112)
Supplement: Supplementary file 1 [file marinedrugs-18-00112-s001.pdf]

Supplementary

Investigating the Antiparasitic Potential of the Marine Sesquiterpene Avarone, its reduced form Avarol, and the Novel Semisynthetic Thiazinoquinone analogue Thiazoavarone

Concetta Imperatore[1,2#], Roberto Gimmelli[3#], Marco Persico[1,2#], Marcello Casertano[1,2], Alessandra Guidi[3], Fulvio Saccoccia[3], Giovina Ruberti[3], Paolo Luciano[1], Anna Aiello[1,2], Silvia Parapini[4], Sibel Avunduk[5], Nicoletta Basilico[6], Caterina Fattorusso[1,2*], and Marialuisa Menna[1,2*]

[1] The NeaNat Group, Department of Pharmacy, University of Naples "Federico II", Via D. Montesano 49, 80131 Napoli, Italy; cimperat@unina.it (C.I.); marco.persico@unina.it (M.P.); marcello.casertano@unina.it (M.C.); pluciano@unina.it (P.L.); aiello@unina.it (A.A.)

[2] Italian Malaria Network, Centro Interuniversitario di Ricerche Sulla Malaria (CIRM), Department of Pharmacy, University of Naples "Federico II", Via D. Montesano 49, 80131 Napoli, Italy;

[3] Institute of Biochemistry and Cell Biology, National Research Council, Campus A. Buzzati-Traverso, Via E. Ramarini, 32, 00015 Monterotondo (Roma), Italy; roberto.gimmelli@ibbc.cnr.it (R.G.); alessandra.guidi@ibbc.cnr.it (A.G.); fulvio.saccoccia@ibbc.cnr.it (F.S.); giovina.ruberti@cnr.it (G.R.)

[4] Dipartimento di Scienze Biomediche per la Salute, Università di Milano, Via Pascal 36, 20133 Milan, Italy; silvia.parapini@unimi.it (S.P.)

[5] Department Vocational School of Medicinal Health Services, Mugla University, Mugla, 48187 Turkey; sibelavunduk@mu.edu.tr (S.A.)

[6] Dipartimento di Scienze Biomediche, Chirurgiche e Odontoiatriche, Università di Milano, Via Pascal 36, 20133 Milan, Italy; nicoletta.basilico@unimi.it (N.B.)

[#] **These authors equally contributed**

* Correspondence: mlmenna@unina.it Tel. +39081678518 (M.M.); caterina.fattorusso@unina.it (C.F.) Tel. +39081678544

TABLE OF CONTENTS

Figure S1. HRESIMS spectrum of thiazoavarone (**2**) 3

Figure S2. ^{1}H NMR spectrum of thiazoavarone (**2**) in $CDCl_3$ 3

Figure S3. ^{1}H-^{1}H COSY spectrum of thiazoavarone (**2**) in $CDCl_3$ 4

Figure S4. HSQC spectrum of thiazoavarone (**2**) in $CDCl_3$ 4

Figure S5. HMBC spectrum of thiazoavarone (**2**) in $CDCl_3$ 5

Figure S6. HRESIMS spectrum of avarone (**1**) 5

Figure S7. ^{1}H-NMR spectrum of avarone (**1**) in $CDCl_3$ 6

Figure S8. HRESIMS spectrum of avarol (**3**) 6

Figure S9. ^{1}H-NMR spectrum of avarol (**3**) in $CDCl_3$ 7

Figure S10. Conformational enantiomers of thiazoavarone (**2**) 8

Figure S11. Calculated pka values of avarol (**3**) (ACD/Percepta software) 9

Figure S12. DFT conformer of **1-3,** and their radical species. 10

Table S1. ΔE_{GM} values and torsion angle values of the DFT conformers of avarol (**3**) 11

Table S2. Hydrogen atoms suitable for an intramolecular radical shift 12

Table S3. cLogD values of **1-3** 13

Scheme S1. Reduction pathway of quinones in a protic solvent 14

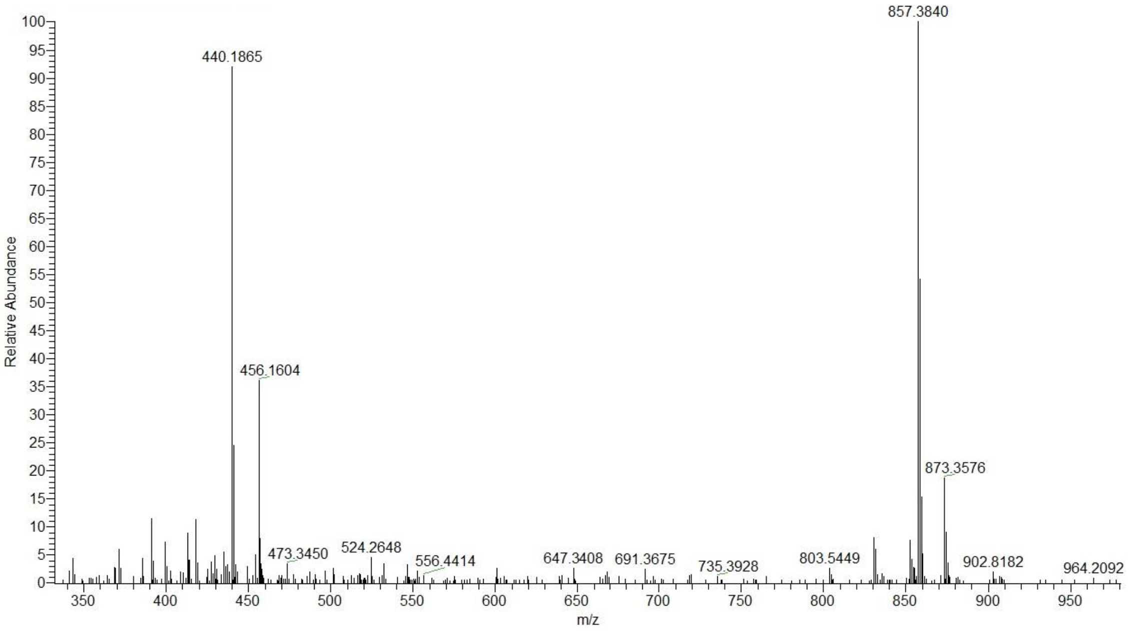

Figure S1. HRESIMS spectrum of thiazoavarone (**2**)

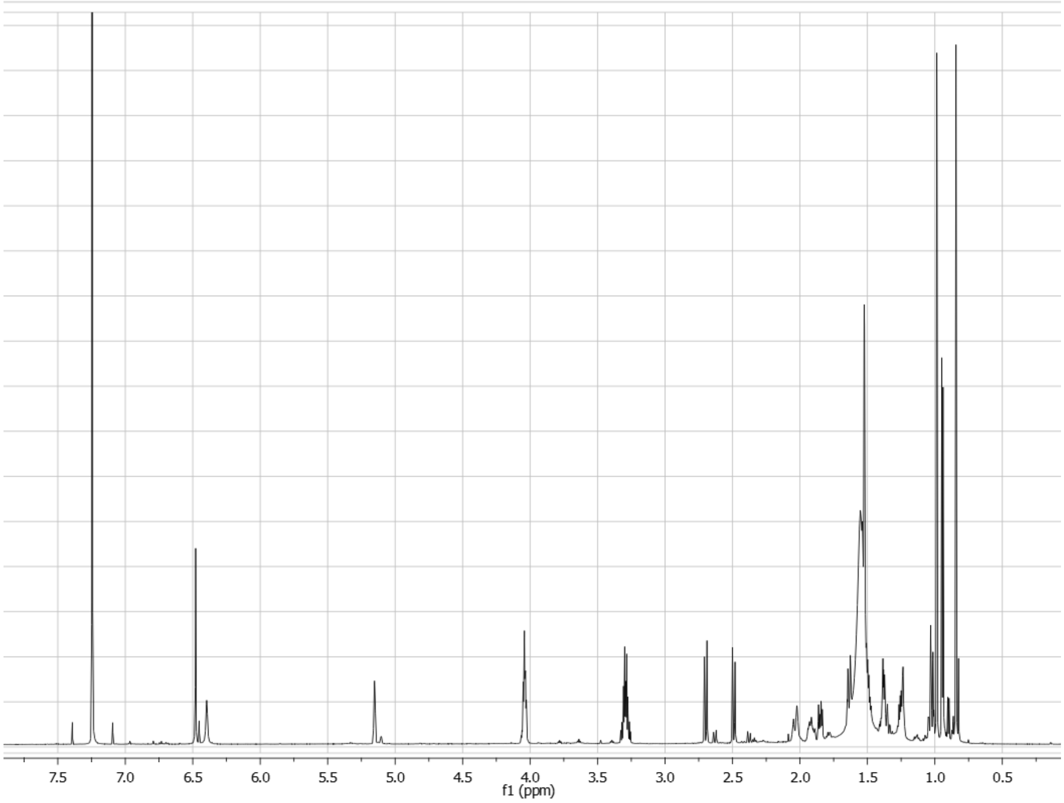

Figure S2. ^{1}H NMR spectrum of thiazoavarone (**2**) in $CDCl_3$

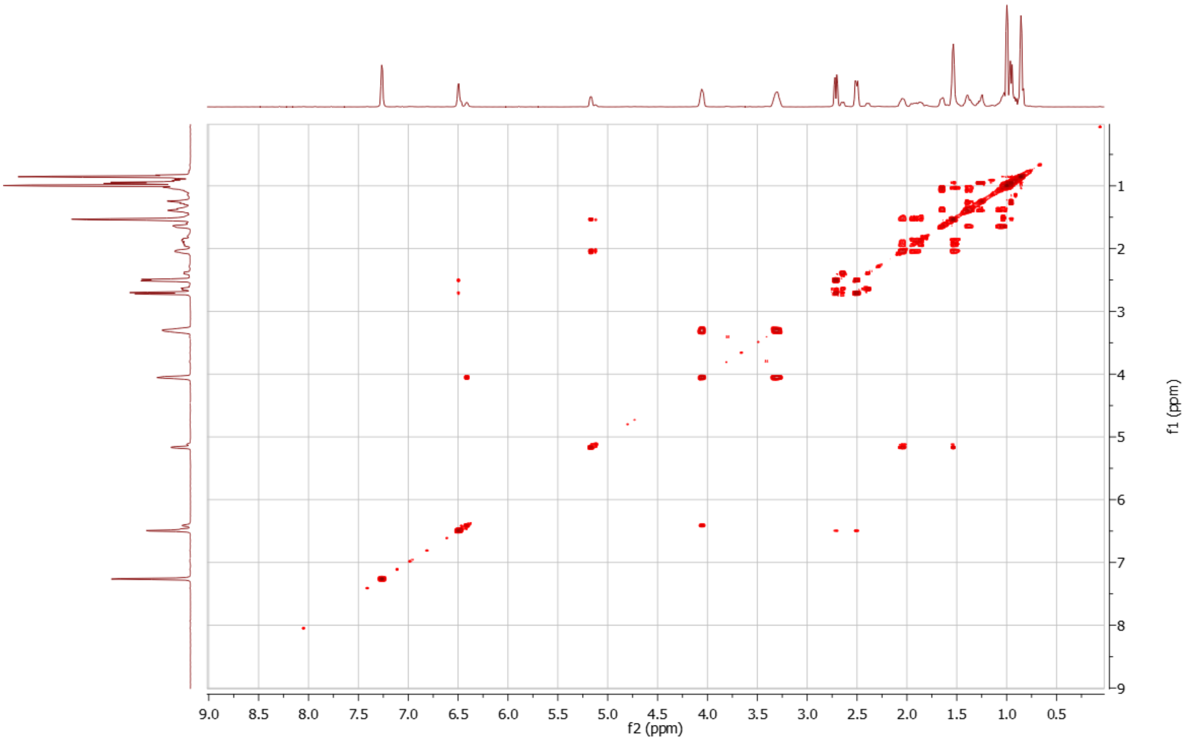

Figure S3. ¹H-¹H COSY spectrum of thiazoavarone (**2**) in $CDCl_3$

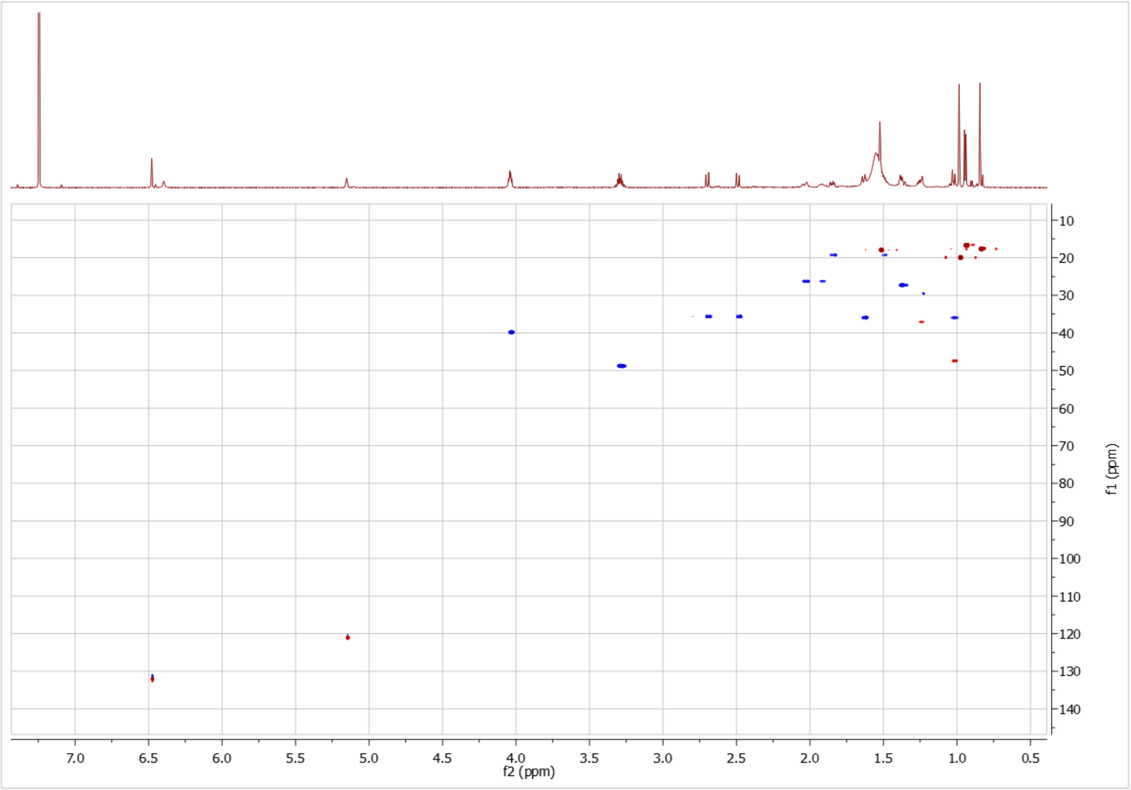

Figure S4. HSQC spectrum of thiazoavarone (**2**) in $CDCl_3$

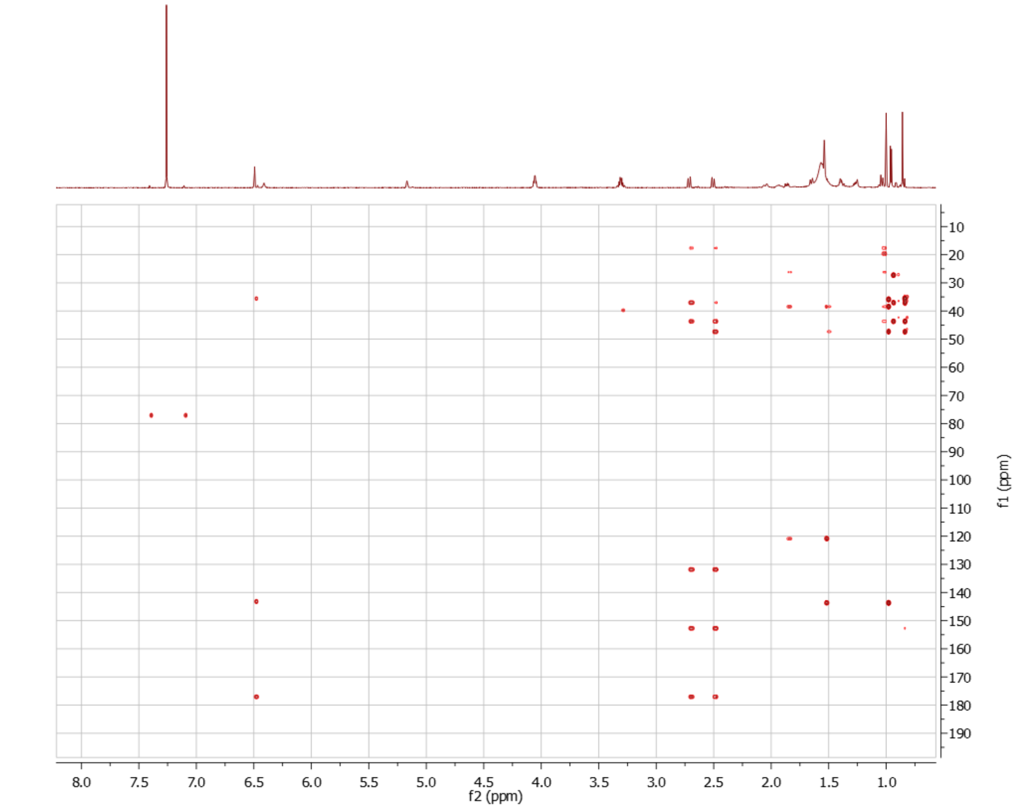

Figure S5. HMBC spectrum of thiazoavarone (**2**) in $CDCl_3$

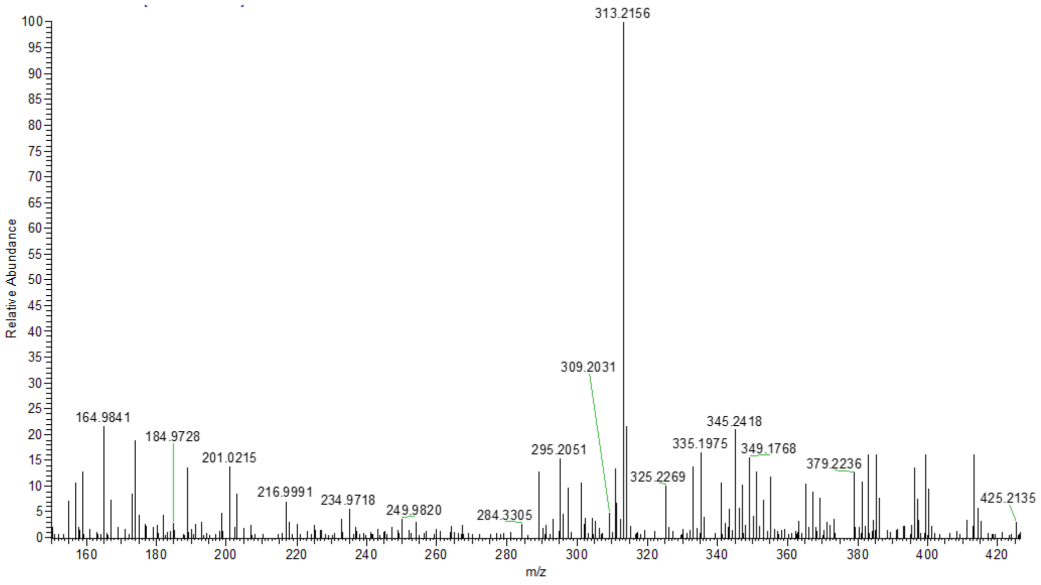

Figure S6. HRESIMS spectrum of avarone (**1**)

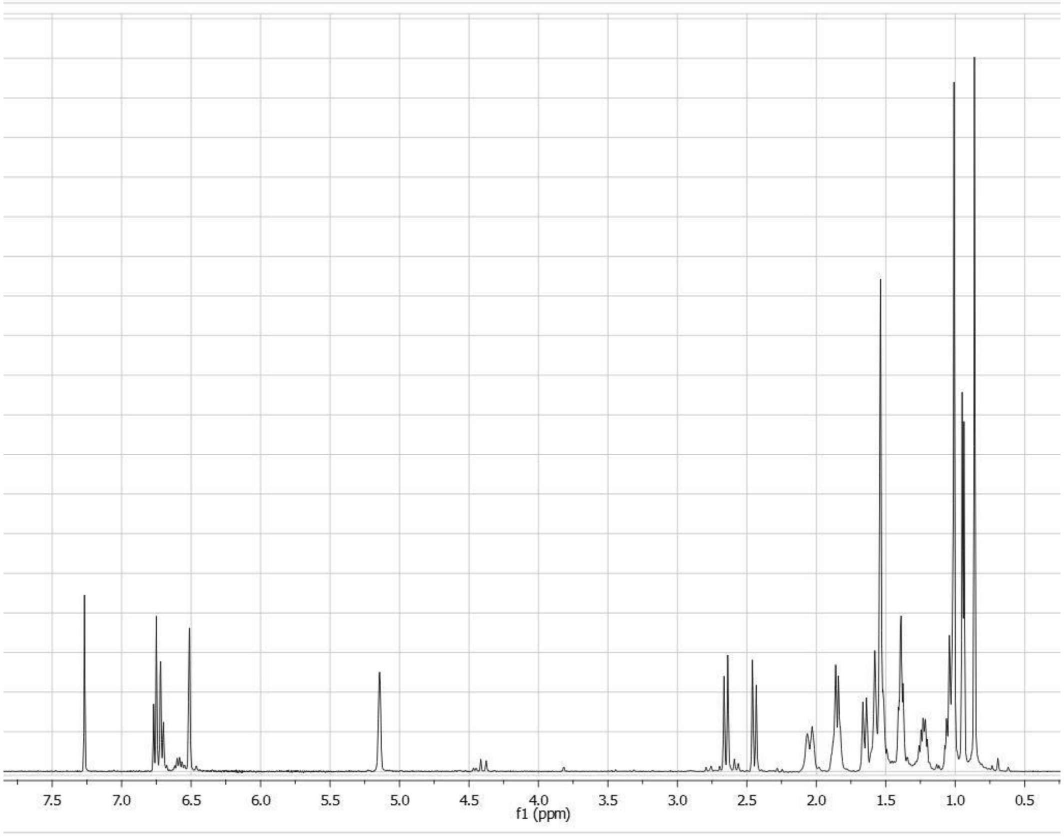

Figure S7. ^{1}H-NMR spectrum of avarone (**1**) in $CDCl_3$

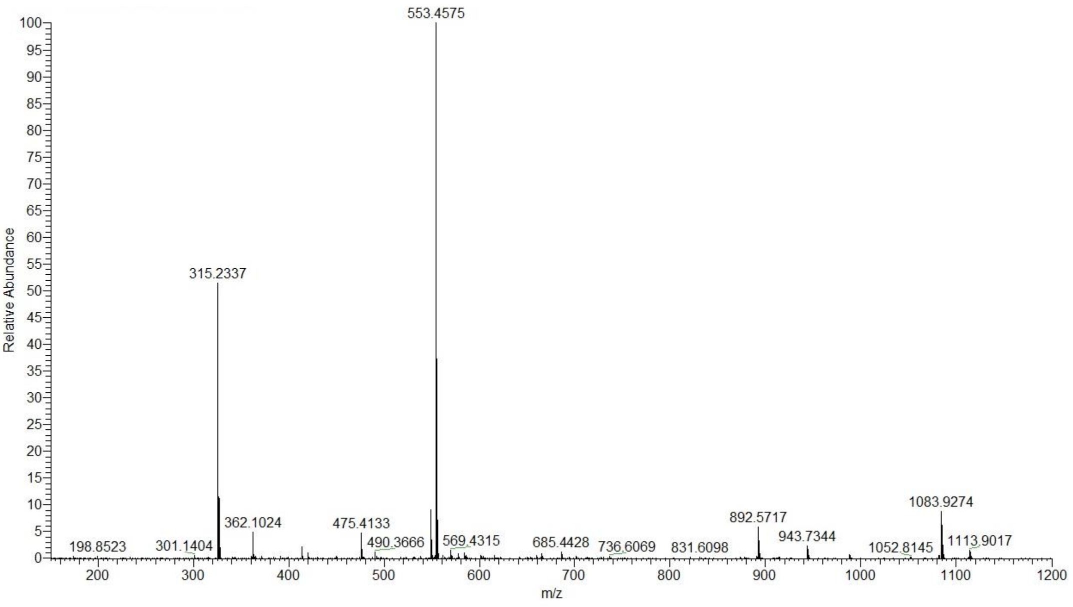

Figure S8. HRESIMS spectrum of avarol (**3**)

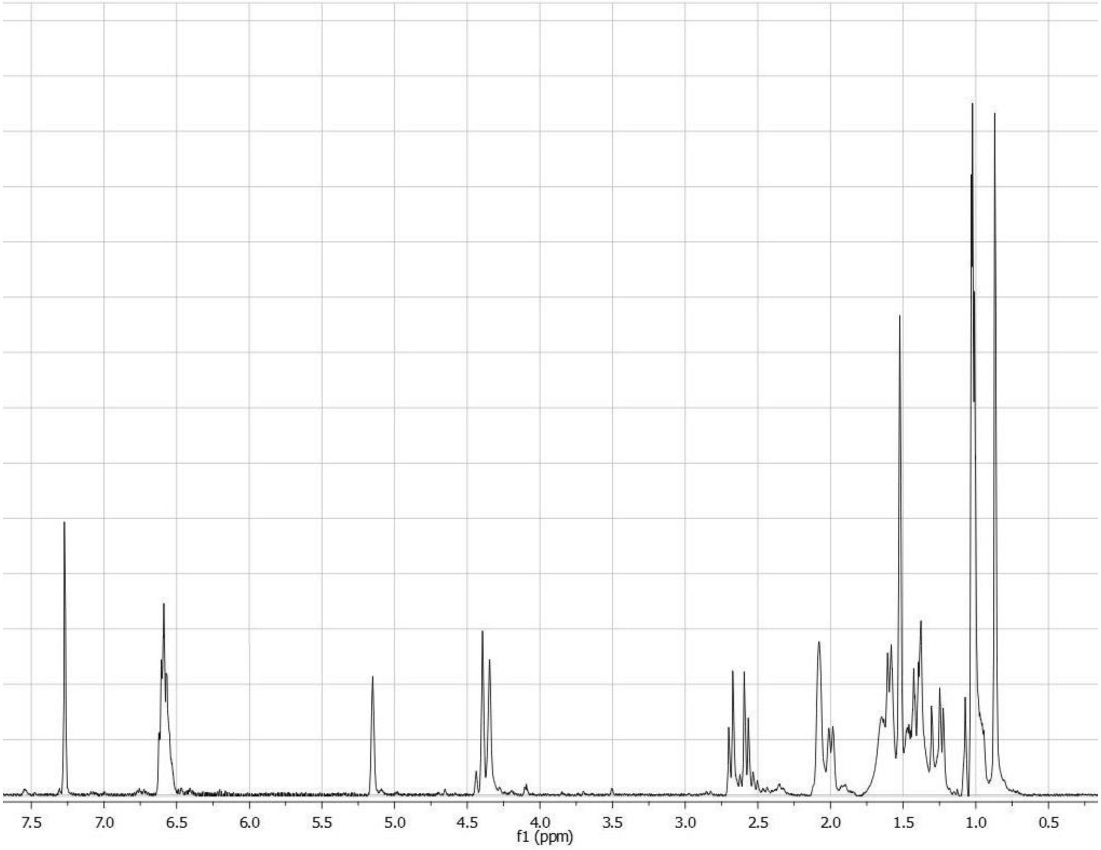

Figure S9. ^{1}H-NMR spectrum of avarol (**3**) in $CDCl_3$

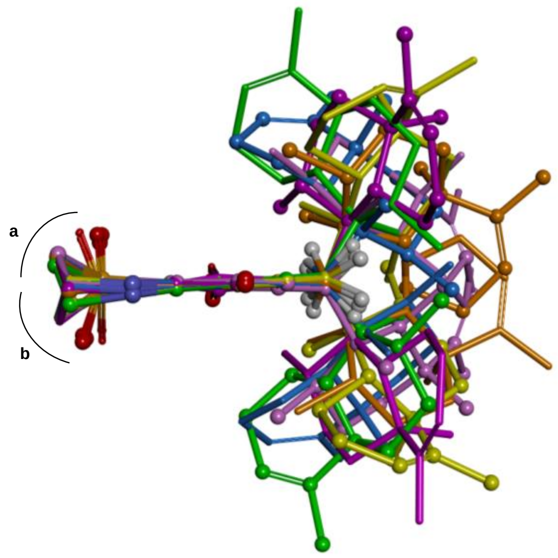

Figure S10. DFT conformers of compound **2** superimposed by the carbon atoms of quinone ring. The conformers characterized by flip a are showed in ball and stick while those characterized by the flip b are showed in stick. Carbon atoms are coloured according to family classification (I=green, II=magenta, III=pink, IV= loght blue, V= orange, and VI=Yellow); heteroatoms are colored by atom type (O= red, N= blue, S= yellow). Hydrogens are omitted for sake of clarity, with the exception of those of the first R′ methylene group.

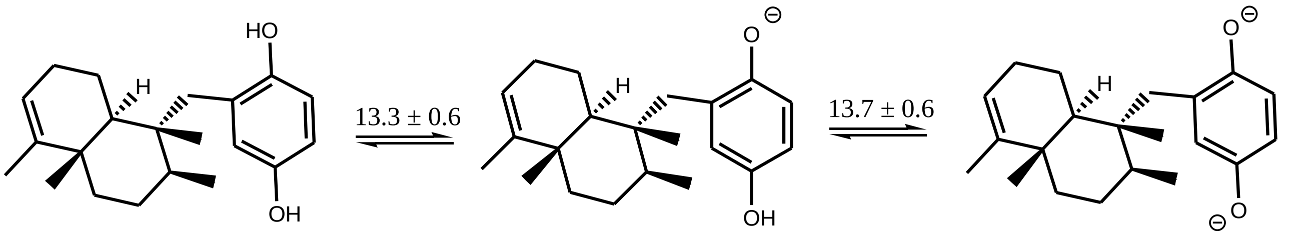

Figure S11. Structures of ionic forms and microcostant pka values calculated for **3** using the algorithm ACD/pKa Classic (ACD/Percepta software).

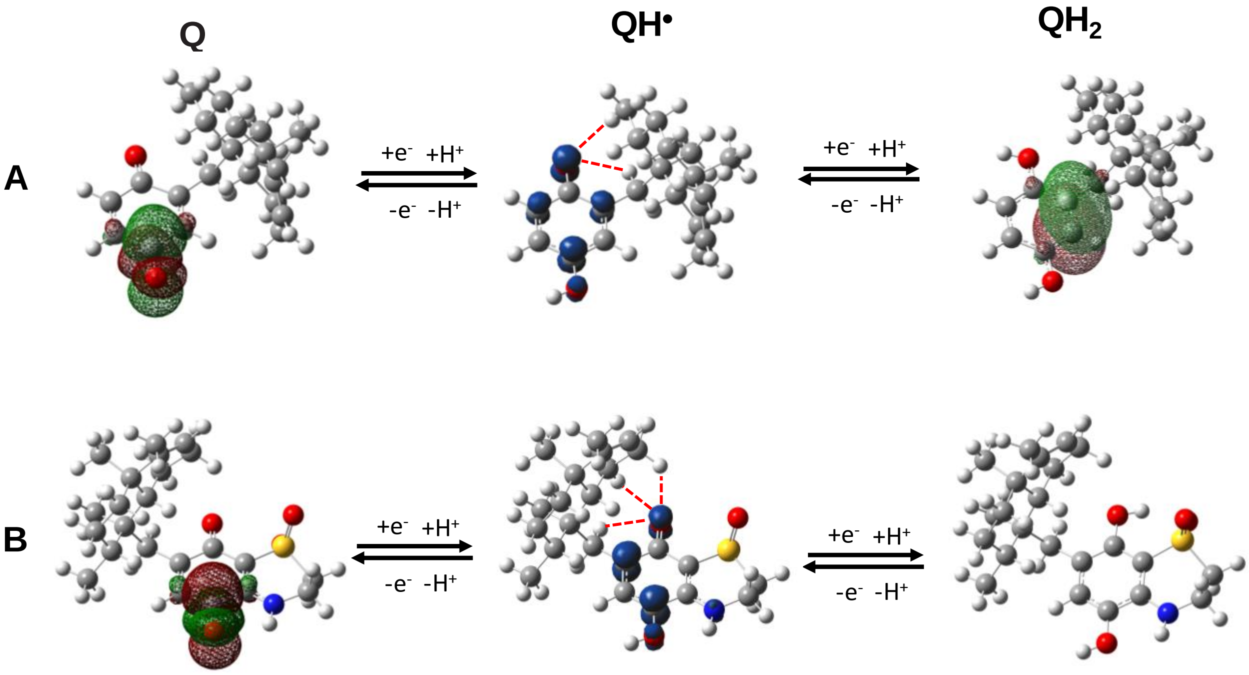

Figure S12. DFT conformer II of **1** (Q,), **3** (QH2) and their semiquinone radical (QH•). (B) DFT conformer I of **2** (Q,) together with its one- and two-electron reduced species QH• and QH2. Atoms possibly involved in an intramolecular radical shift are evidenced with red dashed lines. The LUMO of **1** and **2**, and the HOMO of **3** are visualized using GaussView with an isosurface value of 0.02 $e^-/a.u.^3$ The NBO spin density isosurface of the QH• species is displayed using GaussView with an isosurface value of 0.01 $e^-/a.u.^3$. The blue surface (positive spin density) corresponds to an excess of α-electron density.

Table S1. ΔE$_{GM}$ values (kcal/mol) and torsion angle values (degrees) of the DFT conformers of **3**.

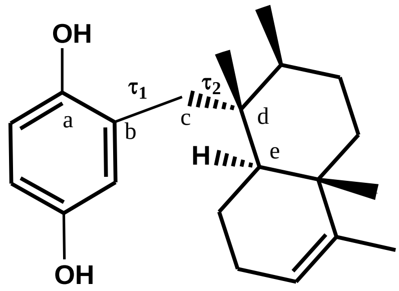

**Conf.**	**ΔE$_{GM}$ (kcal/mol)**	**Torsion Angles (°)**	
		τ1[a]	τ2[b]
**II**	0.00	96.77	56.16
**I**	0.23	-104.40	59.80
**III**	1.45	-102.26	-179.87
**IV**	1.71	108.27	-69.84
**V**	2.12	89.20	179.85
**VI**	2.68	-86.45	-76.24

[a]τ1 torsion angle is defined by a, b, c, and d atoms. [b]τ2 torsion angle is calculated considering b, c, d, and e atoms.

Table S2. List of the hydrogen atoms at a distance suitable for an intramolecular H-radical shift from a carbon atom of the R′ substituent to the reduced/oxidized oxygen atom of the semiquinone radical. Corresponding distances in the Q, $QH^{\bullet}$ and QH_2 forms are also reported. Values are expressed in Å.

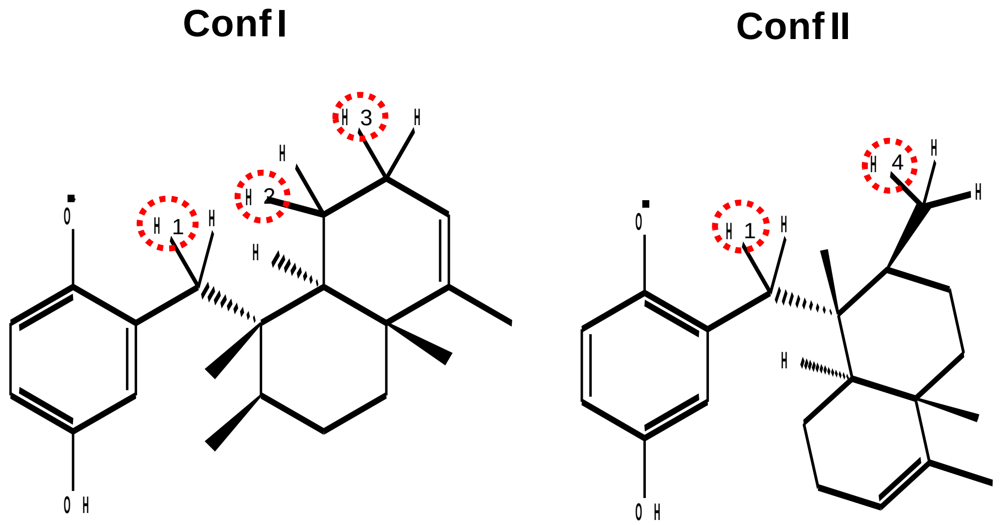

Cmp	Conf	Distance	Q species	$QH^{\bullet}$ species	QH_2 species
**Avarone/Avarol**	I	O-H1	2.43	2.41	2.43
	I	O-H2	2.45	2.40	2.45
	I	O-H3	2.92	3.02	2.77
	II	O-H1	2.43	2.42	2.46
	II	O-H4	2.49	2.45	2.54
**Thiazoavarone**	I	O-H1	2.35	2.39	2.41
	I	O-H2	2.39	2.39	2.44
	I	O-H3	3.04	3.02	2.82
	II	O-H1	2.38	2.40	2.45
	II	O-H4	2.47	2.47	2.56

Table S3. ClogD of compounds **1-3**.

Cmp	cLogD[a]
**1**	6.13
**2**	3.83
**3**	5.22

[a]logD calculated considering pH values: 7.4, 7.2 and 5.5.

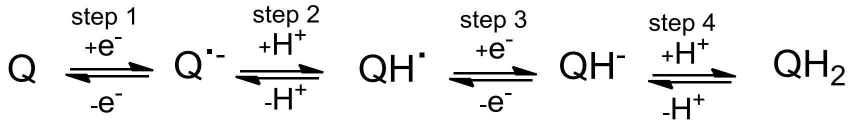

Scheme S1. Reduction pathway of quinone to hydroquinone in aqueous environment.
